# Breast Milk Oligosaccharides Contain Immunomodulatory Glucuronic Acid and LacdiNAc

**DOI:** 10.1016/j.mcpro.2023.100635

**Published:** 2023-08-18

**Authors:** Chunsheng Jin, Jon Lundstrøm, Emma Korhonen, Ana S. Luis, Daniel Bojar

**Affiliations:** 1 Proteomics Core Facility at Sahlgrenska Academy, University of Gothenburg, Gothenburg, Sweden; 2 Department of Chemistry and Molecular Biology, University of Gothenburg, Gothenburg, Sweden; 3 Wallenberg Centre for Molecular and Translational Medicine, University of Gothenburg, Gothenburg, Sweden; 4 Department of Medical Biochemistry and Cell Biology, University of Gothenburg, Gothenburg, Sweden

**Keywords:** glycan, mass spectrometry, glycomics, biodiversity, carbohydrates

## Abstract

Breast milk is abundant with functionalized milk oligosaccharides (MOs) to nourish and protect the neonate. Yet we lack a comprehensive understanding of the repertoire and evolution of MOs across Mammalia. We report ∼400 MO-species associations (>100 novel structures) from milk glycomics of nine mostly understudied species: alpaca, beluga whale, black rhinoceros, bottlenose dolphin, impala, L'Hoest's monkey, pygmy hippopotamus, domestic sheep, and striped dolphin. This revealed the hitherto unknown existence of the LacdiNAc motif (GalNAcβ1-4GlcNAc) in MOs of all species except alpaca, sheep, and striped dolphin, indicating the widespread occurrence of this potentially antimicrobial motif in MOs. We also characterize glucuronic acid-containing MOs in the milk of impala, dolphins, sheep, and rhinoceros, previously only reported in cows. We demonstrate that these GlcA-MOs exhibit potent immunomodulatory effects. Our study extends the number of known MOs by >15%. Combined with >1900 curated MO-species associations, we characterize MO motif distributions, presenting an exhaustive overview of MO biodiversity.

## Highlights

• Profiled milk glycomes of nine understudied mammalian species *via* LC-MS/MS.
• Discovered >100 novel glycan sequences.
• Uncovered widespread existence of LacdiNAc and glucuronic acid motifs.
• Glucuronic acid-containing oligosaccharides are potent immunomodulators.
• Curated dataset constitutes an exhaustive overview of milk glycan biodiversity.

## Graphical Abstract

**Figure:**
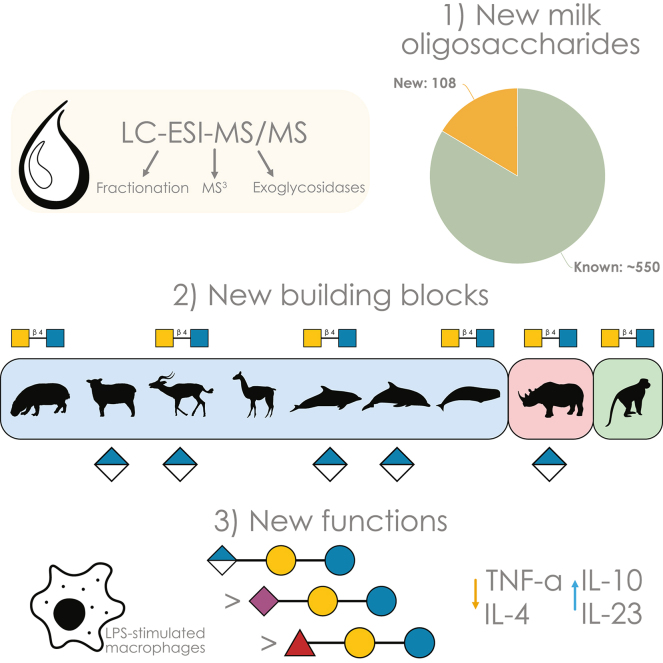

## In Brief

Jin *et al.* deeply profile the free milk glycomes of nine understudied mammalian species. They uncover >100 novel structures that include unusual motifs, such as the widespread use of LacdiNAc and glucuronic acid. This expands known milk oligosaccharide diversity by >15%. Functional experiments indicate potent immunomodulatory effects of novel glucuronic acid–containing milk oligosaccharides. Through contextualization with literature data, they provide an exhaustive overview of milk glycan biodiversity.

While every mammalian species produces breast milk to nurture its young, there is a pronounced diversity in breast milk composition (1). This ranges from the evolutionarily ancient monotreme milk over marsupial milk, designed to nourish the prematurely born neonates in the pouch (2), up to placental milk, including humans and their domestic animals. This broad range of needs and niches has to be recapitulated on the molecular level to endow the respective milk with its required functionality, adjusted during the time after parturition.

Regardless of milk origin, complex carbohydrates are usually among the most abundant components of breast milk (3, 4). Most milk oligosaccharides (MOs) contain a lactose core at the reducing end, which is further extended with the addition of a β1,3 and/or β1,6-linked *N*-acetylglucosamine (GlcNAc), followed by the addition of β1,3 and/or β1,4-linked galactose (Gal). This results in the formation of structures such as lacto-*N*-tetraose (LNT), lacto-*N*-neotetraose (LNnT), lacto-*N*-hexaose (LNH), and lacto-*N*-neohexaose (LNnH), serving as major core structures that are then further functionalized. The elongation may, for instance, occur with several degrees of polymerization of *N*-acetyllactosamine (LacNAc). These MOs are then further decorated with fucose (Fuc) and/or sialic acid and other glycomotifs. Many aspects of infant nutrition (5), immune system development (6), microbiome development (7), and pathogen defense (8) have all been directly tied to MOs and their biochemical properties. Evolutionarily, the initial function of MOs seemed to have been defensive, with a subsequent expansion into nutrition (9). Due to this plethora of functions, the relative absence of complex MOs from infant formula harbors consequences for infant health and microbiome development (10, 11, 12).

In humans, hundreds of unique MOs have been described (13). While this is often used to imply human exceptionalism, milk from well-investigated domestic animals, such as cows or pigs, exhibits similar orders of magnitude in terms of unique MO structures (14, 15). More importantly, the milk glycomes of different species are characteristic (16, 17, 18), supplying a molecular repertoire to fulfill the functions of breast milk in that species. Usually, motifs within whole glycan structures are functional determinants (19). Therefore, (species-specific) sequence-to-function relationships exist in MOs and are strictly limited by the available motif repertoire.

These MO repertoires are produced in the mammary gland by an array of glycosyltransferases, enzymes starting from lactose or lactosamine in the Golgi apparatus, and consecutively adding monosaccharides to the non-reducing end (20). While MO biosynthesis, especially in non-human species, is still incompletely resolved, an emerging consensus describes evolutionarily conserved MO repertoire patterns in various taxonomic groups (21, 22). A classic example can be found in marsupials, with characteristic oligo-galactose motifs in their MOs that are not found in that form and in abundance in other mammalian groups (21).

However, only a small fraction of mammalian species has been characterized regarding their MOs, with a particular emphasis on humans and domestic animals/model organisms. Further, due to the structural complexity of MOs, studies investigating MO repertoires that rely on comparison to standards or regular mass spectrometry might be restricted to measuring only simpler/already characterized MOs, as pointed out before (13, 23). In contrast, it has been convincingly shown that the usage of appropriate techniques, such as porous graphitized carbon-based chromatography - tandem mass spectrometry (PGC-LC-MS/MS), can result in high-resolution structures of milk oligosaccharides (24, 25). Particularly for characterizing new MO structures, tandem mass spectrometry, including probing with specific exoglycosidases, is often necessary (26) yet infrequently applied when characterizing non-model species. This underestimates the true biodiversity of MOs throughout Mammalia because when these methods are applied high structural resolution can be achieved (16).

Here, we use structural glycomics *via* PGC-LC-MS/MS to characterize the milk glycomes of nine species that have been barely, or not at all, investigated regarding their MOs. We identify 393 MO-species associations in this endeavor, including 108 glycan sequences that have not been described before (supplemental Table S1), out of 172 characterized sequences. We also present the discovery of new motifs for MOs, such as the LacdiNAc motif (GalNAcβ1-4GlcNAc), expanding the known motif repertoire for MOs. We further discover several glucuronic acid (GlcA)-containing MOs in multiple species, previously only identified in cows in recent work (27). Next to expanding the motif repertoire, we therefore also broaden the monosaccharide repertoire in MOs. On human immune cells, these GlcA-MOs demonstrate immunomodulatory effects that seem more potent than established immunomodulators such as 2-fucosyllactose (2′-FL). We contextualize our findings within a newly curated dataset of 1902 MO observations in >100 species, constituting an exhaustive overview of our current knowledge about MO biodiversity. Engaging in various sequence and motif analyses, we reveal clusters of motif usage in mammalian MOs. We envision our findings to substantially extend the known biodiversity of MOs and improve our understanding of the biochemical properties and functions of these important constituents of breast milk.

## Experimental Procedures

### Literature Dataset Curation

To generate a comprehensive resource of all currently known milk oligosaccharides, we manually inspected all 19,902 published articles on PubMed (term = milk+carbohydrate) between 1898 and December 2021. MOs were extracted from papers with sequence information on species-specific milk oligosaccharides. This was supplemented by various Google searches for publications that are not indexed on PubMed (*e.g.*, “milk oligosaccharides”, “milk glycans”, “mammalian breast milk”). We also compared and completed our dataset with MOs from GlyCosmos (28), GlyConnect (29), GlyGen (30), and GlycoStore (31). All MOs were formatted into IUPAC-condensed nomenclature and paired with their full taxonomic information. This resulted in 1902 species-specific MO records from 168 species (with 62 species having only been reported to contain lactose in their breast milk). All curated MOs can be found in supplemental Table S2 and stored within our Python package, glycowork (32).

### Breast Milk Samples From Uncharacterized Mammals

Milk samples from alpaca (*Lama pacos*) and L'Hoest's monkey (*Allochrocebus lhoesti*) were donated by Benoît Quintard from the Zoo Mulhouse (France). Samples from impala (*Aepyceros melampus*) were provided by Thierry Petit from the Zoo La Palmyre (France). Breast milk from pygmy hippo (*Choreopsis liberiensis*) and black rhinoceros (*Diceros bicornis*) was donated by Florine Wedlarski from Zoo de Doué la Fontaine (France). Milk from beluga whale (*Delphinapterus leucas*), bottlenose dolphin (*Tursiops truncatus*), and striped dolphin (*Stenella coeruleoalba*) were provided by Jose Luis Crespo from Fundación Oceanogràfic de la Comunitat Valenciana (Spain). Milk from striped dolphins stemmed from beached individuals. Sheep milk (*Ovis aries*) was gifted by Rykets Gård (Sweden). Thus, collected milk specimens included one non-human primate (L'Hoest's monkey), four non-domesticated herbivores (alpaca, impala, pygmy hippo, and black rhinoceros), one domesticated herbivore (sheep), and three marine mammals (beluga whale, bottlenose dolphin, and striped dolphin). All milk samples constituted mature breast milk, sampled according to local regulations, with an additional colostrum sample from black rhinoceros and a pre-milk sample from a pygmy hippo.

### Milk Oligosaccharide Extraction

**Fig. 1:**
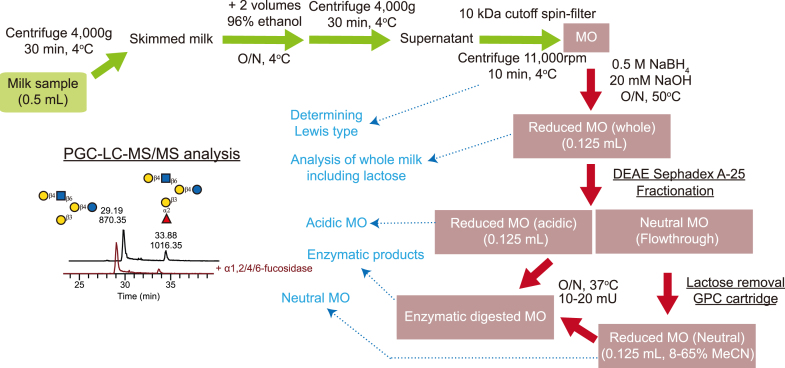
**Schematic workflow of the sample processing workflow used in this work.** For all our milk samples, we started with an initial volume of 0.5 ml milk and proceeded to remove milk fat and proteins. The purified milk oligosaccharides were then used for fractionations, enzymatic digestions, and analysis *via* LC-MS/MS and MS^3^. All mass spectrometry experiments used reduced MOs as a starting material, except for the specification of Lewis antigen types which were assigned by comparison to literature diagnostic ions from underivatized MOs (see Experimental Procedures).

All breast milk samples followed the same protocol (Fig. 1). Per sample, 500 μl of breast milk was diluted with the same amount of distilled water and centrifuged at 4000*g* for 30 min at 4 °C. The only exception stemmed from the hippo pre-milk which was very diluted and started from 70 ml (see below for enrichment details). The skimmed milk was separated from the milk fat layer and the cell pellet and transferred to a fresh tube. Then, two volumes of cold 96% ethanol were added to one volume of milk and incubated at 4 °C overnight to precipitate protein. Subsequently, the sample was again centrifuged at 4000*g* for 30 min at 4 °C. The milk oligosaccharide-containing supernatant was then transferred into a fresh tube, frozen, and lyophilized. Despite the minute starting volume, dictated by very limited sample availability, this was sufficient for all characterization and enzymatic treatment as described in this study.

Freeze-dried milk oligosaccharides were suspended in water (250 μl/ml original milk). Trace proteins were removed by spin-filter (10 kDa cutoff, 11,000 rpm for 10 min, Sigma-Aldrich). The reduction was carried out with 0.5 M NaBH_4_ and 20 mM NaOH at 50 °C overnight. The samples were desalted using cation exchange resin (AG50WX8, Bio-Rad) packed onto a ZipTip C18 tip (Sigma-Aldrich). After drying *via* SpeedVac, additional methanol was added to remove residual borate by evaporation. The samples were then suspended in water (250 μl/ml original milk). The resulting glycans (3 μl per measurement) were then analyzed *via* liquid chromatograph-electrospray ionization tandem mass spectrometry (LC-MS/MS), with or without further fractionation. Relative abundances of experimentally determined MOs can be found in supplemental Table S1.

The released MOs were further fractionated into neutral and acidic fractions using DEAE Sephadex A-25 (GH Healthcare). As lactose is the dominant MO in the neutral fraction, which will suppress the signal of other minor neutral MOs during LC-MS/MS, it was removed using a carbon solid-phase cartridge (HyperSep Hypercarb SPE cartridges 25 ml, Thermo Scientific, Sweden). In brief, the cartridge was conditioned with 3 × 500 μl of 90% MeCN with 0.1% TFA, 3 × 500 μl of 0.1% TFA. After applying the MOs, lactose was eluted with 3 × 500 μl 8% MeCN with 0.1% TFA. The neutral MOs were further eluted with 3 × 500 μl 65% MeCN with 0.1% TFA, dried by centrifugation evaporation, and stored at −20 °C until analysis at a concentration of 250 μl/ml of original milk. To characterize the type of Lewis structure, MOs without reduction were also analyzed.

For 70 ml of *C. liberiensis* pre-milk, we performed affinity chromatography to enrich for GalNAc-terminated MOs, *via* 1 ml of VVL (*Vicia villosa* lectin) linked to agarose beads (AL-1233-2, Vector Laboratories). The beads were incubated with the pre-milk overnight at 4 °C, followed by washing with PBS. Elution was performed by heating the bound material at 70 °C for 1 h (denaturing VVL). Then, we collected the first four fractions for analysis (each fraction equals one column volume or 1.2 ml). All fractions were reduced and desalted as described earlier.

### Analysis of MOs *via* LC-MS/MS

Milk oligosaccharides were analyzed by LC-MS/MS from a stock solution with a sample volume of 250 μl per mL of original milk. The oligosaccharides (3 μl per sample), dissolved in water, were separated on a column (10 cm × 250 μm) packed in-house with 5 μm porous graphitized carbon particles (Hypercarb; Thermo-Hypersil). The oligosaccharides were injected into the column and eluted with an acetonitrile gradient (Buffer A, 10 mM ammonium bicarbonate; Buffer B, 10 mM ammonium bicarbonate in 80% acetonitrile). The gradient (0–45% Buffer B) was eluted for 46 min, followed by a wash step with 100% Buffer B, and equilibrated with Buffer A in the next 24 min.

The samples were analyzed in negative ion mode on an LTQ linear ion trap mass spectrometer (Thermo Electron), with an IonMax standard ESI source equipped with a stainless-steel needle kept at −3.5 kV. Compressed air was used as the nebulizer gas. The heated capillary was kept at 270 °C, and the capillary voltage was −50 kV. A full scan (*m/z* 340 or 380–2000, two micro scans, maximum 100 ms, target value of 30,000) was performed, followed by data-dependent MS^2^ scans (two micro scans, maximum 100 ms, target value of 10,000) with normalized collision energy of 35%, isolation window of 2.5 units, activation q = 0.25, and activation time 30 ms). The threshold for MS^2^ was set to 300 counts. Data acquisition and processing were conducted with Xcalibur software (Version 2.0.7). For comparing MO abundances between samples, individual glycan structures were quantified relative to the total content by integrating the extracted ion chromatogram peak area. The area under the curve (AUC) of each structure was normalized to the total AUC and expressed as a percentage. The peak area was processed by Progenesis QI (Nonlinear Dynamics Ltd).

### Linkage Determination of MOs

MOs were identified from their MS/MS spectra by manual annotation together with exoglycosidase verification. Diagnostic fragmentation ions for the assignment were investigated as described previously (33, 34, 35). For instance, the Lewis types were determined by diagnostic ions resulting from double glycosidic cleavages (C/Z ions) encompassing 4-linked branches obtained from MS/MS of non-reduced MOs. In the case of Lewis a/b, the presence was supported by the fragmentation ions at *m/z* 348, while Lewis x/y was indicated by the fragmentation ions at *m/z* 364 and 510 of underivatized MOs, respectively (35). The cross-ring cleavage (^0,2^A_GlcNAc_-H_2_O) of distal GlcNAc residues was indicative of terminal β1,4-linked Gal (*m/z* 304), GalNAc (*m/z* 425), or GlcA (*m/z* 277; ^0,2^A_Gal_-H_2_O). The ^0,2^X cleavage of sialic acid (*e.g.*, *m/z* 432) is diagnostic of α2,6-linked sialic acid (*e.g.*, Fig. 4*E*). The C/Z double cleavage fragments were used to determine the branching unit (*e.g.*, *m/z* 670 in Fig. 5*C* and *m/z* 549 in Fig. 5*D*).

MOs were treated, prior to re-analysis by LC-MS/MS, with different exoglycosidases, alone or in combination, including α2-3 neuraminidase S (P0743S, NEB, EC number: 3.2.1.18), α2-3,6,8,9 neuraminidase A (P0722S, NEB, EC number: 3.2.1.18), α1-3,6-galactosidase (P0731S, NEB, EC number: 3.2.1.22), β1-4 galactosidase S (P0745S, NEB, EC number: 3.2.1.23), α-*N*-acetylgalactosaminidase (P0734S, NEB, EC number: 3.2.1.49), α1-2,4,6 fucosidase O (P0749S, NEB, EC number: 3.2.1.51), and *E. coli* β-glucuronidase (E-BGLAEC, Megazyme, EC number: 3.2.1.31; desalted and concentrated 10 times with a 10 kDa cutoff spinfilter before use). The recombinant *Bacteroides thetaiotaomicron* 3SGal sulfatase (BT1626, GH2 β-galactosidase) was described previously (36). For enzymatic digestion, we incubated 10 to 20 mU of each glycosidase (except for β-glucuronidase, which we used with a maximum volume) with 15 μl extracted MO at 37 °C overnight in their respective GlycoBuffer as supplied by NEB. The only combinations of enzymes that have been used in this work were sialidase (α2-3 neuraminidase S or α2-3,6,8,9 neuraminidase A) and β1-4 galactosidase S to elucidate the sialylation of type 1 or type 2 LacNAc chains.

Commercial HMOs, including LNT (L403), LnT (L404), DFpLNnH (L807), pLNH (L606), LNFP I (L502), LNFP VI (L514), and LNDFH I (L602), were purchased from Dextra UK. Additionally, 2′-FL (SMB00933-10MG) and 6′-SL (40817-1MG) were obtained from Sigma-Aldrich. Glucuronyl-lactose was obtained from Elicityl Oligotech (GLY180-50%-5MG). These standards were used for generating reference MS/MS spectra and verifying the activity of glycosidases. As glycosphingolipids (GSLs) can share common structures and substructures with MOs, we also used published GSL MS/MS spectra (*e.g.*, Santos *et al.* (37)) as references. The enzymatically treated glycans were desalted as described above. Changes in re-analysis were used for structural elucidation according to the known enzyme specificity. All proposed structures were informed by MS/MS information as well as enzymatic degradation, with example spectra located in Supplementary Dataset 1.

### Immunomodulation Analysis of MOs

Human THP-1 cells (ATCC, TIB-202) were maintained in Roswell Park Memorial Institute 1640 medium (RPMI 1640; Gibco, A1049101) supplemented with 10% (v/v) fetal bovine serum (FBS; Nordic Biolabs, FBS-HI-12A) and 1% (v/v) penicillin-streptomycin solution (Sigma-Aldrich, P4333-100ML) in a humidified atmosphere containing 5% CO_2_ at 37 °C. 4 × 10^4^ cells/well were seeded in a 96-well plate and differentiated into macrophage-like cells by treatment with 25 nM phorbol-12-myristate-13-acetate (PMA; Sigma-Aldrich, P8139-1MG) for 48 h. After exchanging the medium and an additional 24-h rest period, the cells were challenged with 100 ng/ml lipopolysaccharide (LPS; Sigma-Aldrich, L4391-1MG) in the absence or presence of 1 mg/ml chemically pure MOs (2-fucosyllactose: 2′-FL, Sigma-Aldrich, SMB00933-10MG; 6-sialyllactose: 6′-SL, Sigma-Aldrich, 40817-1MG; Glucuronic acid: GlcA, Sigma-Aldrich, G5269-10MG, Glucuronyl-lactose: GlcALac, Elicityl Oligotech, GLY180-50%-5MG). As a control, we also included MO treatment of the cells in the absence of LPS. After 24 h, the culture supernatants were collected and analyzed for the levels of key macrophage cytokines using a multiplex immunoassay based on fluorescence-encoded beads (LEGENDplex; Biolegend, 740503) according to the manufacturer’s instructions. The samples were acquired on an Accuri C6 Plus Flow Cytometer (BD) and the results were analyzed using LEGENDplex Data Analysis Software Suite (Biolegend, Qognit).

### Motif Analysis

All motif analyses were performed using the Python package glycowork (version 0.6.0)^32^. The motif distribution was visualized *via* the *motif.analysis.make_heatmap* function while the linkage patterns were analyzed with the *motif.analysis.characterize_monosaccharide* function. In all analyses, only species with at least five recorded MOs were considered. The clustering of our species was performed by annotating motifs in their MOs *via* the *motif.annotate.annotate_dataset* function, followed by averaging relative abundances across motifs, calculating pairwise cosine distances of species, and hierarchical clustering.

### Experimental Design and Statistical Rationale

Our species selection for this study was strictly limited by sample availability, as access to and availability of exotic mammalian breast milk is extremely scarce. For this, we contacted all major zoological institutions within Europe, as shipping samples from outside Europe would have been severely impeded by CITES regulations. We aimed to obtain as many different samples as possible, within a reasonable timeframe. All statistical tests in this work were done *via* a one-way ANOVA with Tukey's multiple comparison test. In all cases, significance was defined as *p* < 0.05, after correcting for multiple testing.

## Results

### Exploring the Free Milk Glycome of Uncharacterized Mammals

Our extensive literature curation yielded MO data (beyond mere lactose) for 106 species. However, estimates (38) place the total number of mammalian species at about 6,500, suggesting that we only have information for ∼1% of all milk glycomes, and, even where we do have some information, we lack the exhaustiveness applied to human or cow MOs. We thus decided to analyze the free milk oligosaccharides of uncharacterized or undercharacterized mammalian species, using an in-depth structural glycomics approach, to improve overall biodiversity comparisons across Mammalia and expand our knowledge of this important nutrient.

The availability of breast milk from exotic mammalian species is, however, rather limited, which determined our species choice in collaborations with European zoological institutions. This resulted in milk from nine species (seven Artiodactyla, one Perissodactyla, and one Primates), with all samples constituting mature milk (n = 11), and one additional sample of colostrum and pre-milk each. Most of these species had zero previously reported glycans in general and thus add to our knowledge of biodiversity in glycobiology.

We then engaged in an in-depth workflow, in which we combined milk fractionation, enzymatic digestion, and analysis of MOs *via* LC-MS/MS and MS^3^ to aim for a comprehensive characterization of the structure of the MOs in our samples (Fig. 1).

### Common Core of Lacto-*N*-novopentaose I in Non-human MOs

**Fig. 2:**
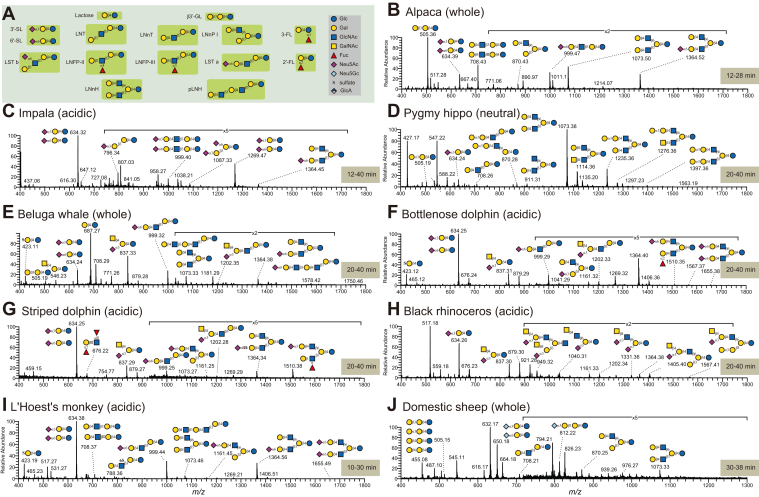
**Typical structures and motifs in free milk oligosaccharides.***A*, examples of common milk oligosaccharide structures, with their commonly used name abbreviations and highlighted motifs, are shown *via* the Symbol Nomenclature For Glycans (SNFG). *B*–*J*, the accumulated MS spectra from different time ranges. To investigate minor and rare MOs in different species, all MOs were separated into neutral and acidic fractions and analyzed by LC-MS/MS separately. With the same purpose, the accumulated MS spectra displayed the MO diversity and complexity. The retention time ranges for each panel are shown in beige boxes. β3′-GL, 3′-galactosyllactose; 2′-FL, 2′-fucosyllactose; 3′-FL, 3′-fucosyllactose; 3′-SL, 3′-sialyllactose; 6′-SL, 6′-sialyllactose; LNFP-II, lacto-*N*-fucopentaose II; LNFP-III, lacto-*N*-fucopentaose III; LNnT, lacto-*N*-neotetraose; LNT, lacto-*N*-tetraose; LST a, sialyllacto-*N*-tetraose a; LST b, sialyllacto-*N*-tetraose b; *p*LNH, para-lacto-*N*-hexaose.

In human MOs (Fig. 2*A*), lactose is the dominant carbohydrate component and major nutrition of infants. In our samples, except for L'Hoest's monkey (5%) and alpaca (21%), the lactose level was indeed much higher than other MOs (56–99%, supplemental Table S1). In our black rhinocerus colostrum sample, this level (78%) was lower than in mature milk (99%) of the same animal. On average, the level of lactose in marine mammals (56–87%) was lower than in terrestrial mammals (77–99%).

**Fig. 3:**
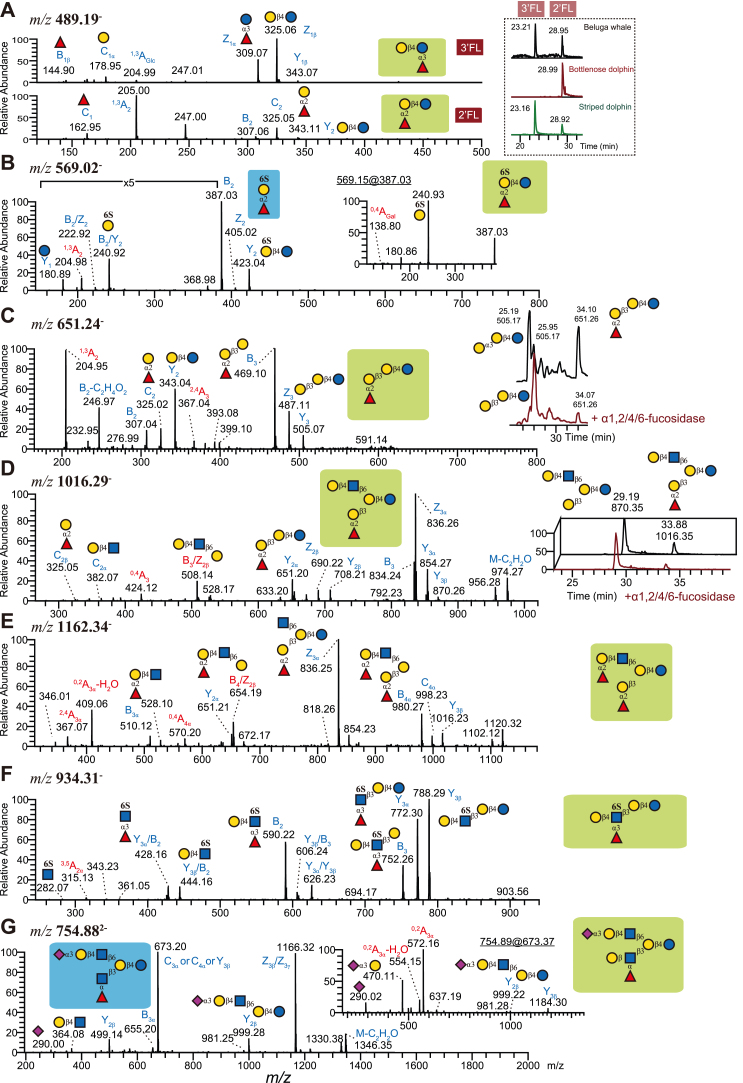
**LC-MS/MS spectra of selected fucosylated MOs.***A*, MS/MS spectra of typical fucosylated lactose (2′-FL and 3′-FL, *m/z* 489, [M-H]^-^). 2′-FL exhibited a longer retention time than 3′-FL on a PGC column. Further, the Z_1***α***_ fragment ion at *m/z* 309 from 3′-FL and C_2_ ion at *m/z* 325 from 2′-FL were two diagnostic ions for these two MOs. *B*, MS/MS spectrum of sulfated 2′-FL detected in bottlenose dolphin. The B_2_ ion at *m/z* 367 and B_2_/Y_2_ ion at *m/z* 241 suggested a sulfated Fuc_1_Gal_1_ motif. The cross-ring cleavage at *m/z* 205 (^1,3^A_2_) indicated ***α***1,2-linked fucose. The insert shows the MS/MS of fragment ions at *m/z* 367. The fragmentation ion at *m/z* 138 (^0,4^A_Gal_) was consistent with a 6SGal. *C*, MS/MS spectrum of fucosylated *β*3′-GL. A series of *A* and *B*/*C* ions indicated a linear Hex_3_ with a terminal ***α***1,2-fucose. After treatment with ***α***1,2/4/6-fucosidase, the accumulated base peak corresponded to *β*3′-GL (see insert). *D* and *E*, MS/MS spectra of fucosylated LNnP-I from bottlenose dolphin. The branching point was determined by the typically dominant B/Z cleavage of surrounding residues around the branching residue, *e.g.*, *m/z* 508 in (*D*) and *m/z* 654 in (*E*). Both Fuc residues were sensitive to digestion by ***α***1,2/4/6-fucosidase. After digestion, the fucosylated structure was converted into LNnP-I. *F* and *G*, MS/MS spectra of MOs containing Lewis x from L'Hoest's monkey and marine mammals. Fragments are annotated according to the Domon and Costello nomenclature (77).

As is the case in human MOs, most MOs here contained a lactose core, for example, LNT, LNnT, or LNnH (Fig. 2, *B*–*J*). However, terminal type 1 LacNAc (Gal***β***1-3GlcNAc) was only detected in L'Hoest's monkey. This is consistent with the observation that type 1 LacNAc is limited to primate MOs. As a consequence, non-primate MOs only exhibited Lewis x motifs and lacked Lewis structures (as described below).

The LNnP-I core (Fig. 2*A*) and elongated MOs of this core are not commonly dominant in human MOs. However, they are a prominent constituent in non-human MOs, including capuchin milk (39). In this study, except for pygmy hippo and impala (both Artiodactyla), all species contained LNnP-I MOs, including L'Hoest's monkey, which were frequently decorated with various motifs, as described below. The enzyme ***β***1,6-*N*-acetylglucosaminyltransferase, which transfers UDP-GlcNAc to 3′-galactosyllactose (***β***3′-GL) and leads to the creation of LNnP-I, was for instance characterized in mammary glands of the tammar wallaby (40) and not detected in human, potentially explaining this MO distribution.

In the following, we will first engage in a general description of the free milk glycome of our investigated species, with the intent of discussing more common elements of their MOs, before we focus on an in-depth characterization of the most novel molecules we discovered herein.

### Exploring the Free Milk Glycomes of Undercharacterized Mammals

In alpaca milk (*L. pacos*, Fig. 2*B*; not studied previously), we detected MOs that were sulfated on galactose or *N*-acetylglucosamine (GlcNAc), including extended structures such as Neu5Acα2-6Galβ1-4GlcNAc6Sβ1-3Galβ1-4Glc (Supplementary Dataset 1, slide 89). Unexpectedly, we only measured two fucosylated glycans, present in low abundance, while sialylated glycans (predominantly Neu5Acα2-6) were more common. This included unusual structures such as Neu5Acα2-3GlcNAcβ1-6Galβ1-4Glc (Supplementary Dataset 1, slide 50), with the rarely observed sialic acid-GlcNAc linkage. Particularly high-abundance MOs were the neutral LNnT (lacto-*N*-neotetraose) and LNnH (lacto-*N*-neohexaose), and the sialylated S-LNnH. Other investigated camelids, *Camelus bactrianus* and *Camelus dromedarius*, differ from this by exhibiting more fucosylated structures, including blood group epitopes (17). Overlaps, such as S-LNnH in *C. bactrianus* and *C. dromedarius* (41, 42), might imply that further investigation of alpaca milk also uncovers these fucosylated MO motifs. We note that *L. pacos* seems to constitute the first example of sulfated MOs in the family of camelids.

Our results from impalas (*A. melampus*, Fig. 2*C*; not studied previously) initially resembled *L. pacos*: lack of fucosylated glycans and presence of sulfated MOs. The most abundant MO, by far, was 3-sialyllactose (3′-SL), followed by 6-sialyllactose (6′-SL). Notably, we identified one lactosamine-based structure, Neu5Acα2-6Galβ1-4GlcNAc, and several structures containing the αGal motif, immunogenic in humans (43). Additional findings included extended sulfated (Gal?1-3Gal6Sβ1-4Glc; Supplementary Dataset 1, slide 22) and phosphorylated (Gal?1-3Galβ1-4GlcP; Supplementary Dataset 1, slide 21) MOs, and highly unusual structures, such as Neu5Acα2-3GlcNAcβ1-6(Galβ1-4)Glc (Supplementary Dataset 1, slide 51). We also detected Neu5Gc as a sialic acid, and a Neu5Acα2-8Neu5Ac motif on one glycan, uncommon on MOs. Several structures, such as Neu5Acα2-3Galα1-3Galβ1-4Glc (Supplementary Dataset 1, slide 47), have, to our knowledge, never been described as a MO and represents a new glycan sequence, sialyl-α3Gl. Other bovines, such as *Addax nasomaculatus* (44), *Bos taurus* (17, 45), or *Bubalus bubalis* (13), share characteristics such as extended sialic acids, usage of Neu5Gc, and lactosamine-based MOs, supporting their conservation in this family.

In the pygmy hippo (*Choeropsis liberiensis*, Fig. 2*D*), the first hippopotamid to be investigated with regard to glycans, we report an abundance of LNnH. *C. liberiensis* expresses both Neu5Ac and Neu5Gc in its MOs and we found several sulfated MOs. Next to an abundance of αGal motifs, other structures of interest included Fucα1-2(GalNAcα1-3)Galβ1-4Glc, exhibiting an A-type blood group antigen, and GalNAcα1-3GalNAcβ1-3Galα1-4Galβ1-4Glc, identified in pre-milk (Supplementary Dataset 1, slide 38 and 63).

Previous research only reported the presence of trace amounts of 3′-SL in beluga whale milk, with even the presence of lactose being questionable (46). This contributed to the impression of a general MO paucity in aquatic animals, with a reliance on milk fats for energy and evolutionary loss of complex MOs (22). However, we characterized 60 unique MOs in beluga whale (Fig. 2*E*), including highly elaborate dodecasaccharides (*e.g.*, Supplementary Dataset 1, slide 172).

Next to the already described 3′-SL, we report several MOs carrying the Sd^a^ epitope (*e.g.*, **Neu5Acα2-3(GalNAcβ1-4)Gal**β1-4Glc; Sd^a^ epitope bold; Supplementary Dataset 1, slide 52), its Neu5Gc-substituted version (Neu5Gcα2-3(GalNAcβ1-4)Galβ1-4Glc; Supplementary Dataset 1, slide 54), and many other extended sialylated structures. Neutral MOs in beluga whales were dominated by LNnT, LNH, and nLc6. Similar to pygmy hippos, relatively closely related to whales, we identified blood group A, αGal, and Lewis x epitopes. Lactosamine-based MOs, sulfated MOs, and Neu5Gc-containing MOs were also detected.

Bottlenose dolphin milk has been tentatively analyzed, with nine reported MO structures (18, 47). Our results, comprising 63 unique structures from two individuals, extend these findings (Fig. 2*F*). Next to the already reported Sd^a^-lactose tetrasaccharide (47), we, for instance, report MOs with two Sd^a^ motifs (Neu5Acα2-3(GalNAcβ1-4)Galβ1-4GlcNAcβ1-6(Neu5Acα2-3(GalNAcβ1-4)Galβ1-3)Galβ1-4Glc; Supplementary Dataset 1, slide 163) or extended a3Gl (Fucα1-2Galα1-3Galβ1-4Glc; Supplementary Dataset 1, slide 31). The dominant neutral structure was LNnT. Other noteworthy motifs include a high degree of fucosylation, Neu5Gc-Sd^a^, blood group A, Lewis x, sulfation, and αGal.

We then analyzed the milk of striped dolphins (Fig. 2*G*; not studied previously). In general, the 45 characterized MOs were reminiscent of bottlenose dolphins, with many Sd^a^ motifs, an abundance of LNnT, and a high degree of fucosylation, including Lewis x structures, DFL, and α2,6-sialylation. Overall, these findings clearly question the presumed MO paucity of aquatic mammals.

For both dolphin species, we analyzed milk from two individuals, revealing interindividual heterogeneity, as some structures were only found in one individual (supplemental Table S1). In well-studied species, such as *B. taurus*, it is known that interindividual differences, as well as differences in lactation time and parity, change the composition of the milk glycome (48), which is likely also the case here. While it can be difficult to obtain a representative set of samples spanning these parameters for species that are not model organisms, our results indicate that this would likely result in many more novel structures in these shallowly investigated animals.

From Perissodactyla, we studied the black rhinoceros, *D. bicornis*, with colostrum and mature milk samples from the same individual. In total, we report 43 unique MO structures (Fig. 2*H*), exceeding the previously reported seven MOs for this species (18), which only contained neutral MOs. Here, we note an abundance of 3′-SL (and an absence of 6′-SL), combined with many MOs containing the Sd^a^ motif. We also note the relative dearth of Neu5Gc-containing MOs in this species. For neutral MOs, LNnP-I and LNH were dominant, and we report several lactosamine-based MOs. In contrast to previous work (18), we did not detect any fucosylated MOs in either sample. We also discovered the new sialyl-α3Gl, first described in impalas, in this sample.

We next investigated the milk from a third taxonomic order, Primates. In our analyzed primate, L’Hoest’s monkey (*A. lhoesti*, Fig. 2*I*; not studied previously), we report extended sulfated MOs and several fucosylated and sialylated structures, predominantly Neu5Acα2-6, and one lactosamine-based structure, Neu5Acα2-6Galβ1-4GlcNAc. After lactose, the most-abundant MO was LSTb (sialyl-lacto-*N*-tetraose b), and we additionally detected a sulfated variant of LSTb with lower abundance. Goto and colleagues (49) also reported sulfated MOs in another Old World monkey, *Papio hamadryas*, and the production of LSTc in *P. hamadryas* and *Macaca mulatta*. However, the finding of LSTb expression and lactosamine-based MOs in *A. lhoesti* seem to be unique among Old World monkeys, for now.

Finally, we turned to domestic sheep, *O. aries*, as a relatively common model system, to assess whether the breadth of structures we uncovered in our more exotic species was also recapitulated in more established species. We note that analyzing this previously characterized type of milk also served as an internal control, as to whether our workflow would recover the known structures from the literature. In total, we characterized 21 unique MO structures in mature sheep milk (Fig. 2*J*), indeed recapitulating all major MOs identified in previous work (16) and extending it by structures such as disialyl-lactose (Neu5Acα2-8Neu5Acα2-3Galβ1-4Glc). This confirmed the robustness of the workflow employed here.

### Fucosylation and Sialylation in MOs of Marine Mammals

Fucosylated neutral human MOs account for 35 to 51% of total MO when excluding lactose (50), with the major fucosylated MO, 2′-fucosyllactose (2′-FL), accounting for up to 6% in secretors. In this study, MOs from marine mammals contained high levels of fucosylated MOs, particularly our two dolphin species (21–31%; Fig. 8*F* and supplemental Table S1), which is why they will be the primary focus in this section. Similar to most isomers in our samples, 2′-FL and 3′-FL were well separated by retention time on our PGC column (Fig. 3*A*, supplemental Table S1). Interestingly, 2′-FL in bottlenose dolphins was dominant and exceeded 20% (Fig. 3*A*, supplemental Table S1). This finding contrasted with a previous study, showing 3′-FL to be dominant in bottlenose dolphins (18), likely due to a different timepoint during lactation. Both beluga whale and striped dolphin, however, exhibited higher 3′-FL than 2′-FL levels in our study (Fig. 3*A*).

**Fig. 6:**
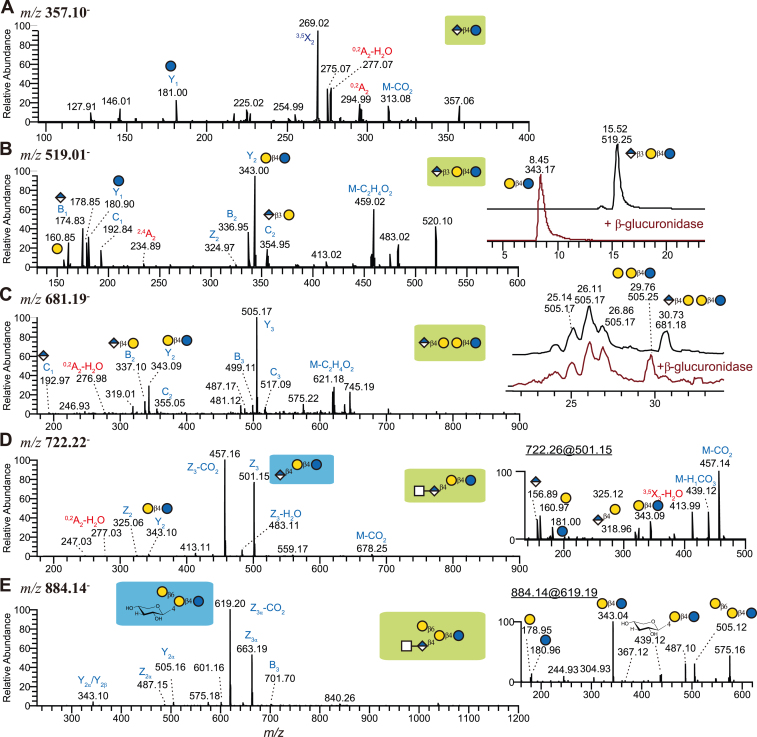
**LC-MS/MS spectra of selected GlcA-containing MOs.***A*, MS/MS spectrum of the shortest GlcA containing MO. The shortest GlcA-containing MO is GlcAβ1-4Glc, which was detected in impala, rhinoceros, and domestic sheep. *B*, MS/MS spectra of commercial standard glucuronyl-lactose (*m/z* 519, [M-H]^-^). The glucuronyl-lactose contains a terminal *β*3-linked GlcA, which can be removed by *β*-glucuronidase (see insert) and converted into lactose (*m/z* 343). The dominant Y/Z and B/C ions are typical fragmentation products of linear oligosaccharides. *C*, MS/MS spectrum of linear GlcA-GL. The GlcA-GL, which was detected in rhinoceros, contained a galactosyl-lactose trisaccharide core. This structure was sensitive to *β*-glucuronidase (see insert). The cross-ring cleavage ions at *m/z* 277 (^0,2^A_Glc_-H_2_O) in (*A*) and ^0,2^A_Gal_-H_2_O in (*C*)) suggested a terminal *β*1,4-linked GlcA. Both MS^2^ (*C*) and MS^3^ spectrum (see insert of *D*) contained fragment ions at *m/z* 413, which were likely a dehydrated ^2,5^X_GlcA_, indicating a potentially 1,4-linked terminal HexNAc. *D* and *E*, MS/MS spectrum of two MOs containing internal GlcA. Both MS/MS spectra are dominated by Z_i_-CO_2_ ions at *m/z* 457 and 619, respectively. The cross-ring cleavage ions at *m/z* 277 indicated a potential *β*4-linked GlcA for the linear one. Furthermore, the fragment ions at *m/z* 439 in the MS^3^ spectrum (see insert of *E*) suggested a branched structure.

**Fig. 7:**
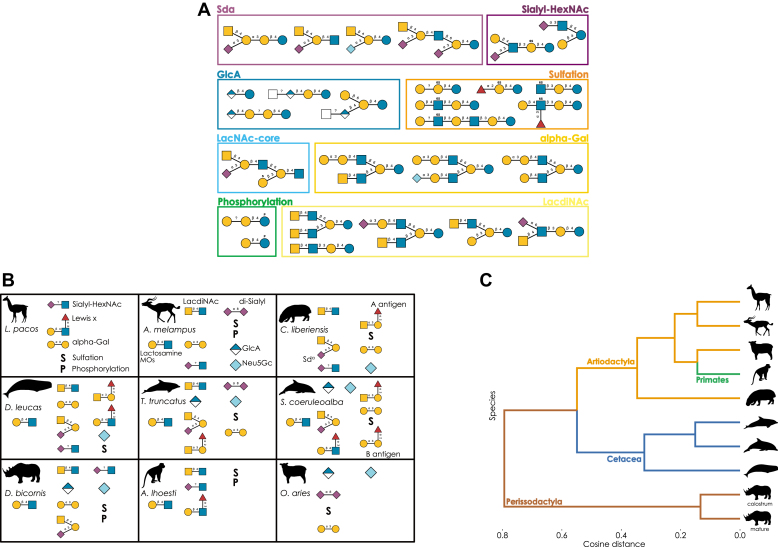
**Newly discovered milk oligosaccharide structures and their taxonomic context.***A*, highlighted new structures. Some of the newly characterized oligosaccharide structures that are reported here are shown as examples of particularly salient glycan motifs in our dataset, shown in the SNFG style. *B*, characteristic milk oligosaccharide motifs. For each newly investigated species, a schematic view of common structural motifs, as identified here, is shown. Structures are depicted using the Symbol Nomenclature for Glycans (SNFG). *C*, clustering of species by their milk glycome. Using the relative abundances from supplemental Table S1, we used glycowork to calculate relative abundances of motifs and converted this into a cosine-based distance matrix of species. This was used for hierarchical clustering, with additional annotation of higher-order taxonomic groups that these species belonged to.

While structures such as 2′-FL are extremely well-researched and widespread, our samples also contained a far greater structural diversity. In total, more than one-third of our herein described fucosylated MOs are first reported in this study (Supplementary Dataset 1, supplemental Table S1), mainly from marine mammals, especially bottlenose dolphins. One example of this can be seen with the novel 6-sulfo-2′-FL structure (Fucα1-2Gal6Sβ1-4Glc; Fig. 3*B* and Supplementary Dataset 1, slide 20), the first fully structurally characterized sulfo-fucosyllactose, a class of MOs that was recently suggested to be present in human milk and may modulate immune activity (51). In general, fucosylation in bottlenose dolphins mainly occurred on terminal Gal residues of LNnP-I (Fig. 3, *C*–*E*). Fragment ions at *m/z* 651 (Y_2***α***_) indicated these as fucosylated ***β***3′-GL, which was sensitive to ***α***1,2/4/6-fucosidase, resulting in the accumulation of ***β***3′-GL (Fig. 3, *C* and *D*). The cross-ring cleavage at *m/z* 409 (^0,2^A_3***α***_-H_2_O) additionally suggested a H type 2 motif on the C6 branch of LNnP-I (Fig. 3*E*).

Several Lewis x containing MOs were detected in L'Hoest's monkey, beluga whale, and striped dolphins. In human MOs, Lewis types are determined by the mother’s secretor and Lewis status. For secretors, Lewis b/y are common MO motifs. However, no Lewis b/y motif was detected in MOs from mammals in this study. As non-human MOs are dominated by type 2 LacNAc chains, the detected Lewis structures in this study contained Lewis x, with or without sulfate (Fig. 3, *F* and *G*).

MOs terminated with blood group A and/or B are common in human MOs and those from carnivorous mammals. In this study, only A-tetrasaccharide (*m/z* 692) and B-tetrasaccharide (*m/z* 651, supplemental Table S1) were observed in MOs from pygmy hippo and marine mammals.

**Fig. 4:**
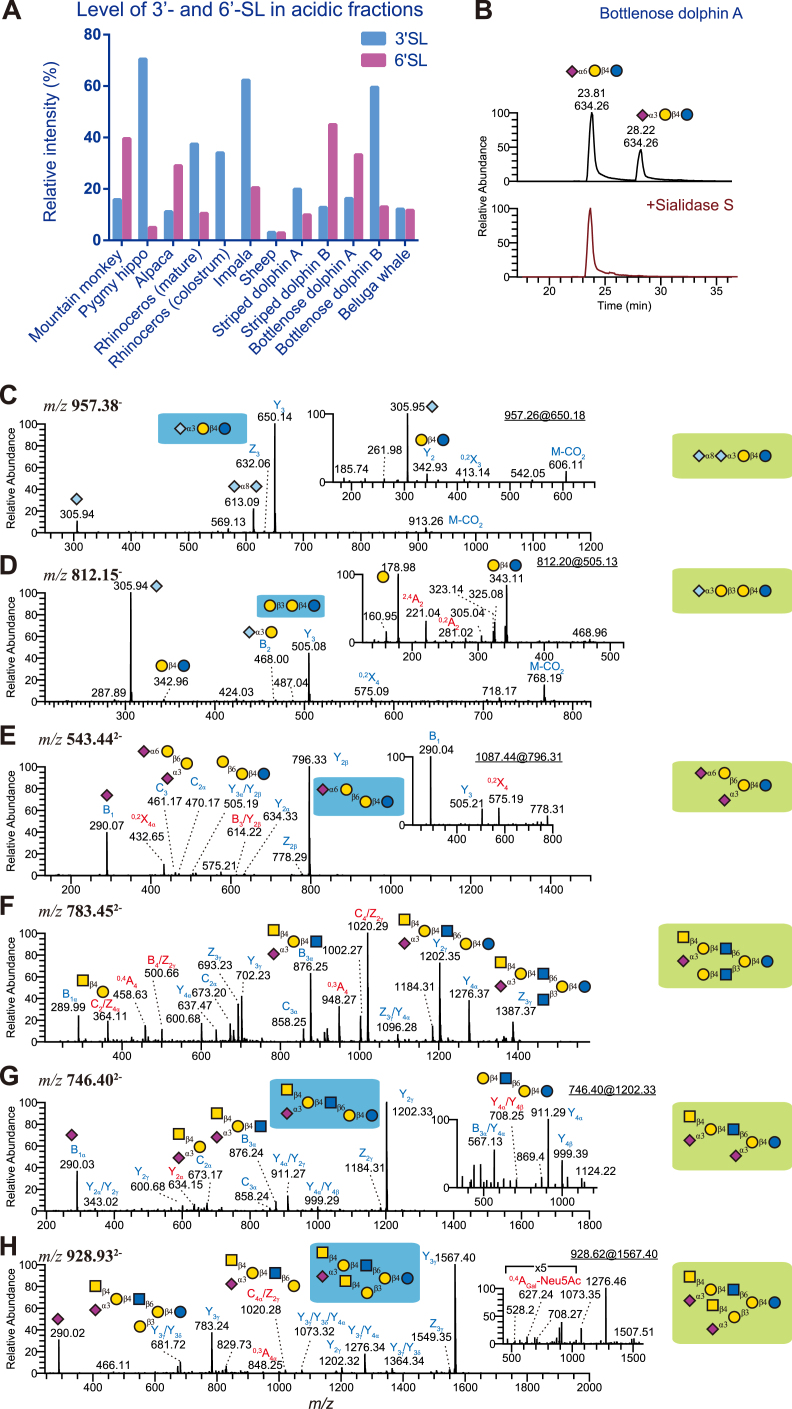
**LC-MS/MS spectra of selected sialylated MOs.***A*, for the acidic fraction of all our milk samples, we assessed the relative abundance, as percent of the total acidic fraction, of 3′-sialyllactose (3′-SL) and 6′-sialyllactose (6′-SL), shown here as paired bar graphs. *B*, enzymatic digestion of sialyllactoses in the acidic fraction of bottlenose dolphin, individual A. The addition of sialidase S led to the disappearance of one peak, which was assumed to contain the *α*2,3-linkage that the enzyme is specific for. Both compounds were also well separated by retention time and, on average, *α*2,3-linked structures elute later on a PGC column than their *α*2,6-linked equivalents (78). *C*, MS/MS spectrum of di-Neu5Gc lactose (*m/z* 957, [M-H]^-^). The B_2_ ions at *m/z* 613 suggested a di-Neu5Gc motif. The insert shows the MS^3^ of fragment ions at *m/z* 650 (Y_3_), which was almost identical to the spectrum produced by 3’-(Neu5Gc)SL. *D*, MS/MS spectrum of sialylated *β*3′-GL (*m/z* 812, [M-H]^-^) in domestic sheep. The presence of B_2_ (*m/z* 468) and Y_3_ ions (*m/z* 505), and the absence of ions indicating terminal Gal (*m/z* 650), suggested a linear trihexoside with a Neu5Gc at the nonreducing end. The insert shows the MS^3^ of fragment ions at *m/z* 505 (Y_3_), which resembled the MS/MS of β3′-GL. This is consistent with previous studies (16). *E*, MS/MS spectrum of disialylated *β*6′-GL (*m/z* 543, [M-H]^2-^). The ^0,2^X_4α_ cleavage of sialic acid (*m/z* 432) is diagnostic of *α*2,6-linked sialic acid. C_3_ ions at *m/z* 461 suggested two sialic acid residues linked to a di-galactoside. The insert shows the MS/MS of fragment ions at *m/z* 796 (Y_2β_). The presence of the ^0,2^X_4_ fragment ion in this MS^3^ spectrum enabled us to assign the *α*2,6-linked sialic acid to the *β*1,6-branch. *F*, MS/MS spectrum of Sd^a^-containing LNnH (*m/z* 783, [M-H]^2-^). The fragment ions at *m/z* 1387 (Z_3γ_), together with C_2α_ and C_3α_ ions at *m/z* 673 and 876, respectively, suggested a branched MO. This structure was resistant to sialidase S digestion. Together with the presence of C/Z ions at *m/z* 364 and 1020, this indicated a branched MO with a terminal Sd^a^ epitope on the C6 branch. *G*, MS/MS spectrum of Sd^a^-containing and sialylated iso-LNnT (*m/z* 746, [M-H]^2-^). The presence of C_2α_ and B_3α_ ions at *m/z* 673 and 876, respectively, together with the resistance to sialidase S digestion, indicated this structure to also be terminated with a Sd^a^ epitope. The insert shows the MS/MS of fragment ions at *m/z* 1202, confirming a sialylated iso-LNnT. *H*, MS/MS spectrum of di-Sd^a^ containing LNnP-I. The C/Z ions at *m/z* 1020 indicated a branched MO. The insert shows the MS/MS of fragment ions at *m/z* 1567, which was dominated by the sialylated C6 branch. The fragment ions at *m/z* 627 were assigned as a ^0,4^A cleavage of the Gal residue of the lactose core, confirming a C6 branch with a Sd^a^ epitope.

Sialylated MOs have been repeatedly shown to be particularly important due to their ability to act as soluble decoys preventing pathogen and toxin invasion (8). Both ***α***2,3-and ***α***2,6-linked sialic acid were detected in the MOs of all our species (Fig. 4*A*). Digestion with ***α***2-3/6/8-specific and ***α***2,3-specific sialidases (Sialidase S, Fig. 4*B*), together with diagnostic ions of individual MS/MS spectra, yielded Neu5Ac linkage information.

In humans, 6′-SL is dominant in colostrum but declines during lactation, similar to 3′-SL (52). Our only primate, L'Hoest's monkey, exhibited a higher level of 6′-SL (Fig. 4*A*). All non-domesticated herbivores, except for alpaca, contained higher levels of 3′-SL, especially in the colostrum of rhinoceros (Fig. 4*A*). This is consistent with other studies with non-domesticated herbivores, for example, yak, buffalo, and camel (52). The amount of 3′- and 6′-SL in the acidic fraction of domestic sheep milk was the lowest (ca. 3%), due to the exclusive sialylation with Neu5Gc instead of Neu5Ac (45% for 3′-GL and 24% for 6′-GL, supplemental Table S1), consistent with a prior analysis of domestic sheep milk (16).

A small fraction of di-sialylated lactose with two Neu5Ac and/or Neu5Gc (*m/z* 925, 941, and 957 in Fig. 4*C* and supplemental Table S1) was detected in impala (only di-Neu5Ac form) and sheep. No fucosyl-sialyllactose was detected in any sample. We further identified various sialylated structures with a trisaccharide core (Fig. 4, *D* and *E*).

The addition of the Sd^a^ blood group on glycoproteins and glycolipids occurs in humans and other animals. In this study, Sd^a^-containing MOs were detected in several species, including all three marine mammals. Previously, Sd^a^-lactose tetrasaccharide was the only reported Sd^a^-containing MO in the milk of bottlenose dolphins (39). Among our 63 characterized structures in that species, 10 contained one or more Sd^a^ motifs, including highly elaborate structures (Fig. 4, *F*–*H*). The Sd^a^ motifs mainly decorated the terminal Gal residues of LNnP-I in our two dolphin species (*e.g.*, Fig. 4*H*).

In agreement with previous work (16), the level of Neu5Gc was highest in MOs from domestic sheep, with dominant 3′-*N*-glycolylneuraminlyllactose (3′-GL), whereas no Neu5Gc was detectable in L'Hoest's monkey, alpaca MOs, and mature MOs from rhinoceros.

### LacdiNAc is Widespread in MOs of Artiodactyla Species and Beyond

**Fig. 5:**
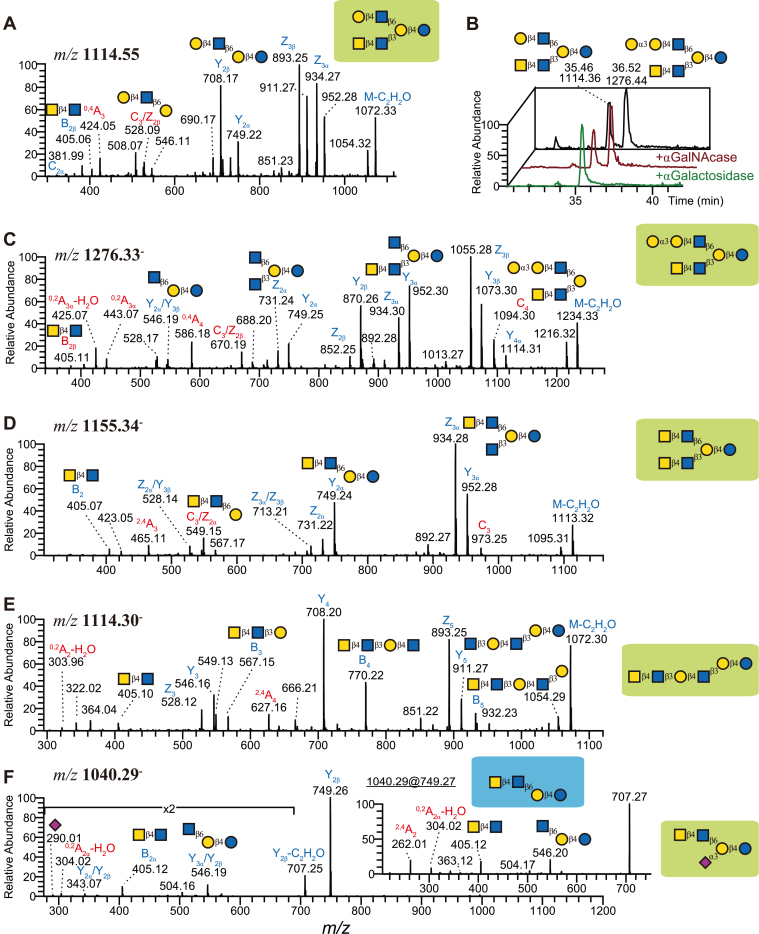
**LC-MS/MS spectra of selected LacdiNAc containing MOs.***A*–*C*, MS/MS spectra and enzymatic digestion of *α*Gal and LacdiNAc substituted LNnH (*m/z* 1276, [M-H]^-^). This structure (*C*) was resistant to chicken liver *α*GalNAcase digestion but sensitive to *α*-galactosidase (*B*). After digestion, this structure turned into LdiNnH (*A*). In both spectra, dominant Y/Z ions indicated the loss of HexNAc and Hex residues from both branches. The LacdiNAc (B_2_ ions) was assigned to the C3 branch due to the presence of ^0,4^A_Gal_ (*m/z* 586 and 424, respectively) and C/Z ions (*m/z* 670 and 528, respectively). *D*, MS/MS spectrum of LNnH substituted with two LacdiNAc moieties (*m/z* 1155, [M-H]^-^). The B_2_ ions at *m/z* 405 suggested the presence of a LacdiNAc motif. The Y/Z ions were associated with the loss of only one or two HexNAc residues. Together with the C/Z ions at *m/z* 549, it revealed two LacdiNAc moieties. *E*, MS/MS spectrum of a linear LacdiNAc-hexasaccharide (*m/z* 1114, [M-H]^-^, para-LdiNnH). A series of B ions at *m/z* 405, 567, 770, and 932 (B_2_ to B_5_) suggested a linear structure, which was clearly different from (*A*). The corresponding cross-ring cleavage ions at *m/z* 304 and 302 (^0,2^A_2_) and 627 (^2,4^A_4_) confirmed it to be a linear LacdiNAc-hexasaccharide. *F*, MS/MS spectrum of sialylated and LacdiNAc substituted LNnT (*m/z* 1040, [M-H]^-^). The fragment ions at *m/z* 405 and 546 suggested a HexNAc_2_-linked lactose. MS^3^ of fragment ions at *m/z* 749 (see inset) confirmed the presence of LacdiNAc.

In a surprising discovery, six of the novel glycans in *A. melampus*, such as GalNAcβ1-4GlcNAcβ1-3Galβ1-4Glc and GalNAcβ1-4GlcNAcβ1-3(Galβ1-4GlcNAcβ1-6)Galβ1-4Glc (Fig. 5*A*; Supplementary Dataset 1, slide 44 and 92), exhibited a terminal LacdiNAc motif (GalNAcβ1-4GlcNAc), an important motif in protein-linked glycans that has not yet been described in MOs. Next to being the most likely biosynthetic explanation for two connected HexNAcs (prominently indicated by fragments at *m/z* 405), we also confirmed this assignment *via* diagnostic ^0,2^A_GlcNAc_-H_2_O ions at *m/z* 304 (*e.g.*, Fig. 5, *E* and *F*), indicating a 1 to 4 linkage. Further, the resistance of these structures to *α*GalNAcase treatment (Fig. 5*B*) suggested a beta-linkage, concluding in our assignment of these structures as *β*1,4-linked LacdiNAc.

The two examples described earlier constituted GalNAc-substituted versions of LNnT and LNnH, and we propose to refer to them as LdiNnT (lacto-*N*,*N*-neotetraose) and LdiNnH. The MS/MS spectrum of LdiNnH containing LacdiNAc on both branches (Fig. 5*D*) contained fragment ions at *m/z* 405, 465, and 549. These were assigned as B_2_ ions (the LacdiNAc moiety), ^2,4^A cleavage of the branching Gal, and the branching Gal with the C6 branch, respectively, confirming the structure assignment. No ions indicated the presence of an alternative linear structure. Thus, this structure was assigned as LdiNnH, containing LacdiNAc on both branches, and only detected in impala. GalNAc in general constitutes a relatively rare monosaccharide in MOs, mostly found in blood group A, and its usage within a LacdiNAc motif exhibits important functions in protein- and lipid-linked glycans (53, 54), likely also indicating a functional role in MOs.

Similar to impalas and pygmy hippos, we found nine LacdiNAc-containing structures in the milk of *D. leucas*. Next to the ones already described above, this included a GalNAc-substituted version of para-LNnH (GalNAcβ1-4GlcNAcβ1-3Galβ1-4GlcNAcβ1-3Galβ1-4Glc; Fig. 5*E*; Supplementary Dataset 1, slide 94), para-LdiNnH in our proposed nomenclature, and sialylated LacdiNAc structures.

Even in a different taxonomic order, Perissodactyla, we structurally characterized LacdiNAc-based MOs in the milk of black rhinoceros, such as GalNAcβ1-4GlcNAcβ1-6(Neu5Acα2-3)Galβ1-4Glc (Fig. 5*F*; Supplementary Dataset 1, slide 81), also present in beluga whales. We further report sialylated LacdiNAc structures, GalNAcβ1-4(Neu5Acα2-6)GlcNAcβ1-3Galβ1-4Glc (Supplementary Dataset 1, slide 82), shared with impalas and beluga whales. In impala milk, this is accompanied by structures such as Neu5Acα2-3(GalNAcβ1-4)GlcNAcβ1-3(Galβ1-4GlcNAcβ1-6)Galβ1-4Glc (Supplementary Dataset 1, slide 120), which is reminiscent of a hybrid between LacdiNAc and the Sd^a^ motif, Neu5Acα2-3(GalNAcβ1-4)Gal. Notably, this can be further functionalized, such as in Galα1-3Galβ1-4GlcNAcβ1-3[GalNAcβ1-4(Neu5Acα2-6)GlcNAcβ1-6]Galβ1-4Glc, an extended structure representing an LNnH backbone with an αGal motif and a sialylated LacdiNAc motif (Supplementary Dataset 1, slide 146).

In total, we identified 10 LacdiNAc-containing MOs, out of 60 characterized structures, in pygmy hippos alone. Sialylation of GlcNAc residues in particular is still insufficiently understood. Even in humans, the responsible enzyme is not yet fully known, with indications for ST6GALNAC6 (55).

We also did detect a LacdiNAc glycan in L’Hoest’s monkey, GalNAcβ1-4GlcNAcβ1-3Galβ1-4Glc or LdiNnT (Supplementary Dataset 1, slide 44). Another Old World monkey, *M. mulatta*, was also reported to exhibit GalNAc in its MOs (49) yet not in the characteristic LacdiNAc motif. We speculate that the LacdiNAc motif might be found in other Old World monkeys as well. The existence of LacdiNAc-exhibiting MOs in the three orders Artiodactyla, Perissodactyla, and Primates, as shown here, suggests that this motif could be conserved in the whole of Mammalia.

### Perissodactyla and Aquatic Artiodactyla Species exhibit GlcA in Their MOs

Another major finding from our impala milk was the presence of glucuronic acid (GlcA) in one glycan (Supplementary Dataset 1, slide 2). Its MS/MS spectrum contained Y_1_ ions at *m/z* 181 and ^0,2^A cross-ring cleavages (*m/z* 295 and 277) of the Glc residue, with the fragment at *m/z* 277 indicating a 1,4-linkage, suggesting the linear disaccharide GlcAβ1-4Glc. Importantly, we found this same molecule also in domestic sheep milk and that from black rhinoceros (supplemental Table S1). Usually, GlcA is found in proteoglycans. Only recently, the first GlcA-containing MOs were reported, albeit not fully structurally characterized, in cow milk, another bovine species, with speculations about their role in immunomodulation (27). We thus set out to further investigate this understudied area of MO biochemical diversity.

First, to confirm the nature and linkage of GlcA in this, and in the glycans following below, we performed exoglycosidase digestion with a commercial *β*-glucuronidase (E-BGLAEC, Megazyme). We first tested this assay on the most suitable commercial standard for glucuronyl-lactose (Elicityl Oligotech, GLY180-50%-5MG), in which GlcA was connected *via* a *β*1,3 linkage to lactose. This compound was indeed sensitive to digestion by this enzyme (Fig. 6*B*), confirming the workflow for GlcA-terminated glycans below. Working with this commercial standard also allowed us to ascertain which fragments do occur in a genuine GlcA-containing MO, including the characteristic B_1_/C_1_ ions for terminal GlcA (*m/z* 175 and 193, respectively).

We then set out to identify extended GlcA-containing MOs. In total, we identified four GlcA-containing MOs in the colostrum of *D. bicornis*, including the abovementioned structure and structures such as an extended trisaccharide GlcAβ1-4Gal?1-?Galβ1-4Glc (Supplementary Dataset 1, slide 36). As we assigned a terminal GlcA to this structure, we could use the abovementioned *β*-glucuronidase structure and indeed found that this compound was sensitive to digestion (Fig. 6*C*), confirming our assignment. Overall, the ^0,2^A_Gal_-H_2_O cross-ring cleavage at *m/z* 277, together with the enzymatic digestion, suggested a *β*1,4-linked GlcA. Due to the low amount of this structure, the linkage of the underlying tri-saccharide could not be fully characterized, yet it notably appeared at a different retention time than the other tri-saccharides measured in this sample.

Further, in rhinoceros and striped dolphins, we identified another GlcA-containing MO, HexNAc?1-?GlcAβ1-4Galβ1-4Glc (Fig. 6*D*; Supplementary Dataset 1, slide 43). The MS/MS spectrum was dominated by Z_3_-CO_2_ ions at *m/z* 457, which were probably generated by loss of the carboxyl group on C6 of the GlcA (33) and indicated an internal GlcA residue. The presence of cross-ring cleavage ions at *m/z* 277, like the others (Fig. 6, *A* and *C*), suggested a 1,4-linked GlcA to lactose.

In rhinoceros, striped dolphin, and bottlenose dolphin milk (in both studied individuals), we also discovered an extended GlcA-containing MO that seemed to be biosynthetically related to the one in Figure 6*D*, with the sequence HexNAc?1-?GlcAβ1-4(Galβ1-6)Galβ1-4Glc (Fig. 6*E*; Supplementary Dataset 1, slide 62). Its MS/MS spectrum was also dominated by Z_3α_-CO_2_ ions at *m/z* 519, indicating an internal GlcA residue. The Z ions associated with a loss of the full C6 carbon indicated a C4 substitution of GlcA, whereas the MS^3^ confirmed the presence of a branched structure, with indicative fragments at *m/z* 439. While we did not detect ions at *m/z* 277 (diagnostic of *β*1,4-linked GlcA), the presence of such ions in all biosynthetic precursors of this structure made a *β*1,4-linkage exceedingly likely and we speculate that the branched nature of this structure makes the formation of the corresponding cross-ring fragment less likely. We note that, outside of proteoglycans, GlcA in an internal position is highly unusual.

### Surveying the Milk Glycomes of Mammalia

Taken together, we clearly show that the complexity of non-human MOs has been underestimated. Of particular importance, we present 108 entirely new glycan sequences (Fig. 7*A*) out of 172 characterized sequences, extending the total number of unique MOs (estimated at 550–600 based on our extensive literature dataset) by >15% with our single study. New sequences, motifs, and monosaccharides, all conserved to at least some degree within Mammalia (Fig. 7*B*), demonstrate that this realm likely still is full of discoveries and surprises, including many family- and order-specific structures (Fig. 7*C*), such as the Sd^a^ motif that is highly enriched in Perissodactyla and Cetacea, or others that are likely to be antimicrobial/antiviral or otherwise functional. The existence of LacdiNAc- and GlcA-containing MOs in multiple glycans, individuals, species, and taxonomic orders also strongly suggests some degree of conservation of these features in MOs.

**Fig. 8:**
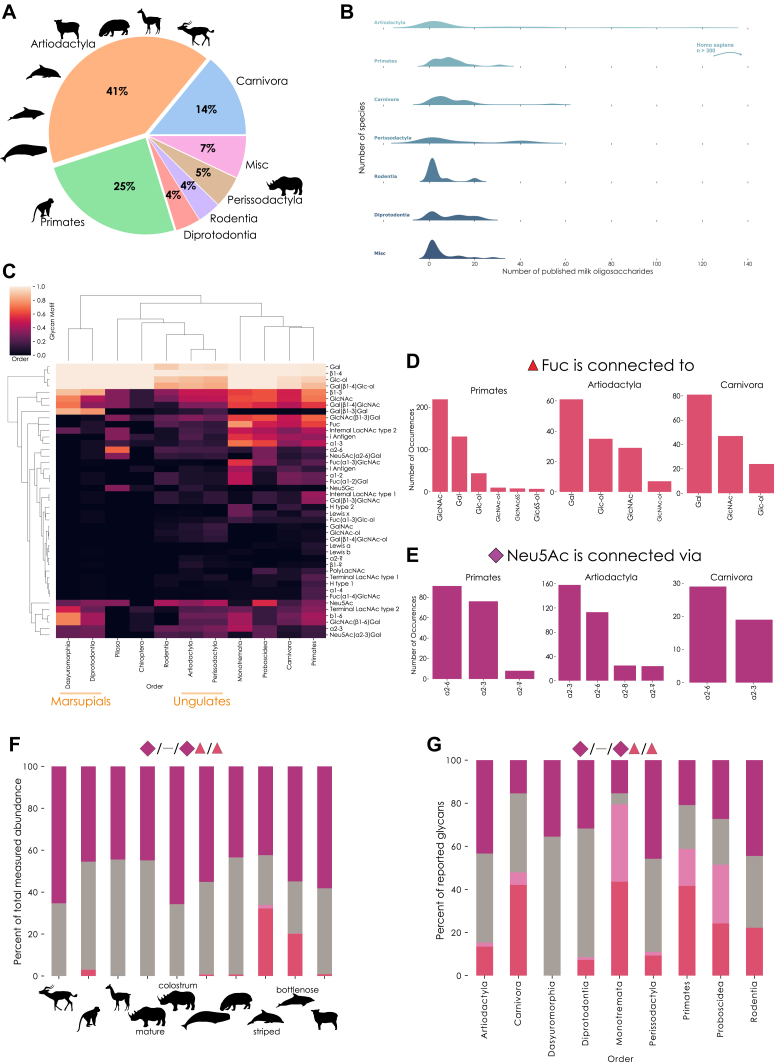
**Properties of all known MOs.***A*, milk oligosaccharides by taxonomic order. A pie chart with the proportion of milk glycans from various mammalian orders is shown, with rare orders being grouped under “Misc”. *B*, glycan distribution by taxonomic order. For each order, the number of milk glycans per species is visualized as a distribution *via* a ridge plot, with the noted exception of the “outlier” *Homo sapiens*. *C*, heatmap of milk glycan motifs. For taxonomic orders with at least five unique known MOs, we analyzed their proportion of motifs using the *annotate_dataset* function of glycowork version 0.6, depicted *via* a hierarchically-clustered heatmap. *D* and *E*, linkage analysis of the monosaccharides fucose (Fuc) and *N*-acetylneuraminic acid (Neu5Ac) in milk glycans. Using glycowork, we analyzed to which monosaccharides Fuc was connected (*D*) and *via* which linkages Neu5Ac (*E*) was found in milk glycans of the three most exhaustively explored taxonomic orders. *F*, proportion of sialylated and fucosylated glycans in our measured species. For each species, we separated glycans into sialylated, fucosylated, fucosialylated, and glycans exhibiting none of these characteristics. For each category, we summed the relative abundances of these glycans and depicted them as a stacked bar graph. *G*, surveying reported sialylated and fucosylated glycans in taxonomic orders. Similar to (*F*), we separated glycans into four sequence groups, but on an order level rather than the species level. We then depicted the fractions of these groups as a stacked bar graph. Note that, while (*F*) depicts relative abundances, this panel depicts the fraction of reported glycans (*i.e.*, presence) with a characteristic.

To gain a sense of the evolution of MOs across mammals, we required a comprehensive dataset, allowing conclusive statements about, for example, motif distributions, according to our current knowledge. Surveying all 1902 (non-unique) published MOs, and combining them with our 393 observations, we find that approximately 80% of known MOs from more than 100 species stem from the taxonomic orders Primates, Artiodactyla, and Carnivora (Fig. 8*A*). These orders contain humans (Primates) and livestock such as cows or goats (Artiodactyla), highlighting the focus on model organisms and commercially/biomedically relevant species, which may not be representative of the number of species per taxonomic order. Even within these orders, coverage per species varies (Fig. 8*B*), from species with lactose as the sole described MO up to the extensively investigated humans. The number of unique MOs per species strongly correlated with the number of publications on the MOs of that species (Spearman’s rank correlation coefficient; ρ = 0.655, *p* < 0.001). This clearly shows that, from a repertoire size perspective, there is little reason to believe that non-human species do not exhibit order-of-magnitude similar MO diversity, making biodiversity mapping crucial to understand the biosynthetic capabilities of Mammalia.

MOs have different sequences, with functional consequences, which also differ by species. Therefore, we sought to characterize the distribution of MO motifs by taxonomic order (Fig. 8*C*). Marsupial orders (Dasyuromorphia and Diprotodontia) clearly separated from other orders, mainly due to a preponderance of neutral MO motifs in marsupials. Other orders, including Primates or Proboscidea, characteristically exhibited fucose- and sialic acid-containing motifs in their MOs, including Lewis blood group antigens.

Focusing on sequence characteristics of the three best-represented orders (Primates, Artiodactyla, and Carnivora), we analyzed their usage of fucose and sialic acids, monosaccharides crucial for anti-pathogenic effects of MOs (56, 57). While fucose was preferentially linked to GlcNAc in Primates, such as in Lewis motifs, we identified galactose as the most frequent neighbor of fucose in MOs of Artiodactyla and Carnivora (Fig. 8*D*). For sialic acid, Primates exhibited approximately the same amount of α2-3 and α2-6 linkages, while Artiodactyla species seemed to prefer Neu5Ac in its α2-3 configuration in their MOs (Fig. 8*E*). We also analyzed post-biosynthetic MO modifications, uncovering widespread sulfation of monosaccharides at C6 in Artiodactyla, Primates, and Rodentia, while sulfation at C3 was largely restricted to marsupials (Diprotodontia/Dasyuromorphia) and, to a lesser extent, Carnivora (supplemental Fig. S1).

In our own collected data, we identified a high degree of sialylation, with fucosylated glycans only being prevalent in the two dolphins and the monkey species (Fig. 8*F*). This, and the general rarity of fucosialylated structures, was also captured in our wider literature dataset (Fig. 8*G*). Here, we identified a larger proportion of fucosylated structures in Primates and Carnivora, while orders such as Artiodactyla and Perissodactyla were characterized by prominence in sialylated structures.

### GlcA Milk Oligosaccharides are Potent Immunomodulators

Building on previous speculations regarding the immunomodulatory effects of GlcA (27), we set out to test the effect of various MO formulations on macrophage activation by LPS. For this, we added physiologically relevant concentrations of chemically pure individual MOs to differentiated THP-1 macrophages – unstimulated and stimulated with LPS – and assessed the response *via* a multiplex cytokine assay.

In human milk, 2′-FL concentrations reach 5 mg/ml in colostrum (for secretors), while median levels decrease to 1 to 2 mg/ml post-partum (58). 6′-SL ranges between 0.05 to 1 mg/ml, highest at parturition. In contrast, the total oligosaccharide content of bovine milk is 10- to 20-fold lower, with 2′-FL and 6′-SL constituting less than 1% respectively (14). Instead, structures such as 3′-SL, 6′-SLN, and 6′-galactosyllactose comprise the major bovine MO content. While physiological concentrations for GlcA-containing MOs are harder to assess, previous studies in bovine milk have found the total of GlcA-containing MOs to reach up to 10% of the total MOs by abundance, depending on the time of lactation (27), which is comparable to the abundance range of sulfated MOs.

**Fig. 9:**
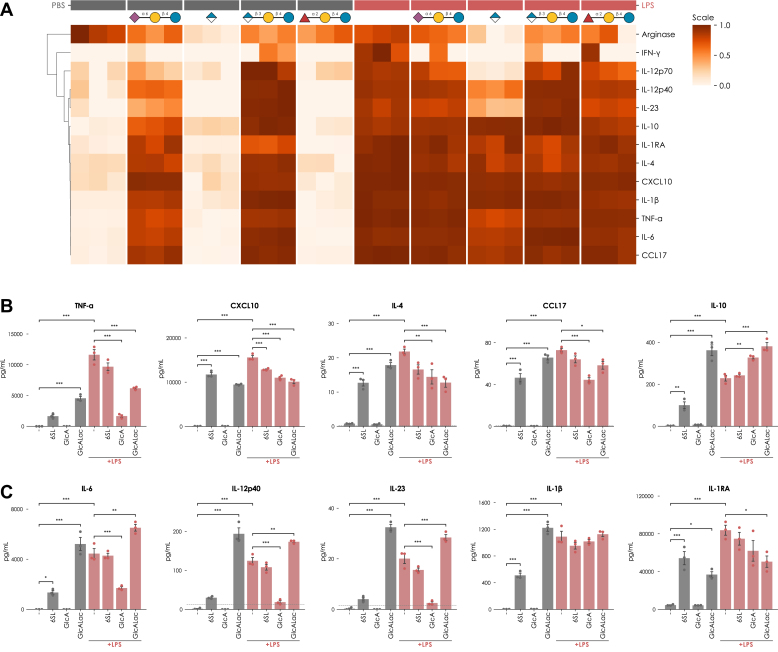
**Glucuronylated milk glycans are immunomodulatory.***A*, heatmap of cytokine concentrations of Arginase, IFN-γ, IL-12p70, IL-12p40, IL-23, IL-10, IL-1RA, IL-4, CXCL10, IL-1β, TNF-α, IL-6, and CCL17 from the culture supernatant of THP-1 cells unstimulated (*grey*) or stimulated with LPS (*red*) in the absence or presence of various MO-derived glycan structures. The row dimension was normalized and clustered by correlation distance. *B* and *C*, Quantification of cytokine concentration of TNF-α, CXCL10, IL-4, CCL17, IL-10, IL-6, IL-12p40, IL-23, IL-1β, and IL-1RA from the culture supernatant of THP-1 cells unstimulated (*grey*) or stimulated with LPS (*red*) in the absence or presence of various MO-derived glycan structures. The dashed line indicates the limit of detection as determined by the standard curve of each analyte. Significant differences were established *via* a one-way ANOVA with Tukey's multiple comparison test. ∗∗∗*p* < 0.001; ∗∗*p* < 0.01; ∗*p* < 0.05.

For 11 out of 13 measured cytokines, LPS treatment significantly upregulated cytokine production compared to baseline levels (Fig. 9, *A*–*C*, supplemental Fig. S2*B*), indicating robust immunostimulation of the macrophages without significant impact on cell viability (supplemental Fig. S2*A*).

In the absence of LPS stimulation, treatment with 2′-FL modulated arginase production but had no impact on the remaining macrophage-derived cytokines (Fig. 9*A*, supplemental Fig. S3). In contrast, treatment with acidic Mos—6′-SL and GlcALac—robustly induced cytokine production in a largely dose-dependent manner, with the GlcALac-induced levels even reaching those achieved by LPS treatment for IL-10, IL-6, IL-12p40, IL-23, and IL-1β. Interestingly, this induction was not observed for the treatment with glucuronic acid as a monosaccharide, indicating the importance of the lactose core structure for its effect (Fig. 9, *B* and *C*, supplemental Figs. S4–S6).

Supporting previous reports on immunomodulatory functions of MOs (59) and GlcA-derived compounds (60, 61), we observed significant inhibition of LPS-induced production of pro-inflammatory cytokines. The GlcA monosaccharide alone displayed an inhibitory effect in the presence of LPS (Fig. 9, *B* and *C*, supplemental Fig. S5). Further, across the range of assayed concentrations, compared to 6′-SL, GlcALac had a more potent effect in dampening the LPS-induced production of Th1-directing molecules, TNF-α and CXCL10, as well as Th2-directing molecules, IL-4 and CCL17, in addition to a more potent induction of anti-inflammatory IL-10 (Fig. 9*B*, supplemental Figs. S4 and S6). Moreover, and in contrast to 6′-SL, high-dose GlcALac treatment further enhanced the LPS-induced production of Th17-inducing cytokines, IL-6, IL-12p40, and IL-23, while also promoting IL-1β signaling through downregulating its inhibitor, IL-1RA (Fig. 9*B*). Interestingly, low-dose treatment with GlcALac significantly inhibited the LPS-induced production of IL-12p40, IL-23, and IL-1β, suggesting a concentration-dependent mechanism regulating the activation-inhibition balance of GlcALac (supplemental Fig. S6). Taken together, these results demonstrate a potent immunomodulatory effect of GlcA-MOs and could indicate a physiological function, such as regulating the infant response to gut-colonizing bacteria through promoting the polarization of IL-10-dependent non-pathogenic Th17 cells (62, 63).

## Discussion

To obtain a comprehensive overview of the biodiversity and evolution of breast milk oligosaccharides, a representative set of Mammalia needs to be studied. However, even though we present and analyze data from a set of over 100 mammalian species, large swaths of Mammalia still constitute frontiers in MO research. Taxonomic orders such as Chiroptera, Cingulata, Didelphimorphia, Eulipotyphla, Lagomorpha, Peramelemorphia, Pilosa, Rodentia, Sirenia, or Tubulidentata are poorly characterized or not at all, regarding their MOs. Including diverse environments, from the sea to the air, we expect many more unexpected biodiversity discoveries in their MOs, akin to the MO LacdiNAc motif uncovered here. Even already characterized species may deserve a revisit as evidenced by our discovery of GlcA-MOs in sheep milk. Additionally, MO descriptions of most exotic mammals rely on a single study and a single population, sometimes even a single individual and/or a single time point during lactation. We are therefore convinced that the diversity and complexity of the various non-human milk glycomes is currently substantially underestimated.

In general, we note that we detected relatively few fucosylated structures, which we ascribe to the rather mature state of most of our milk samples. While our characterization herein relies on negative mode, and thus might slightly overestimate the intensities of sialylated structures, we do not think it likely that this has caused an underestimation of the number of neutral structures. Since we also fractionated all samples into acidic and neutral oligosaccharide fractions, we can be confident that even the neutral fraction of our samples did not contain many fucosylated structures, except for the mentioned dolphin and monkey species.

In other glycan classes, LacdiNAc-decorated glycans, generally more common in non-human animals (64), regulate glycoprotein serum half-life or are involved in tumor progression (65). In HMOs, the LacdiNAc motif seems absent. Aligning with functions of other MO motifs, preventing the adhesion of toxins, bacteria, and viruses by mimicking adhesion epitopes (66), we propose that the herein-discovered LacdiNAc motif in MOs serves a similar purpose for yet to be identified neonate pathogens in LacdiNAc-expressing species. Exhibited by cetaceans, primates, and both ungulates, we envision that the LacdiNAc motif is far from rare in MOs, despite its obscurity to this point. Its biosynthesis remains unclear yet, though it is likely that the isoenzymes B4GALNT3/4, responsible for LacdiNAc synthesis on protein-linked glycans (67), are involved.

Together with recent findings by Gray *et al.*, describing GlcA-containing MOs in cow milk (27), we contribute toward the emerging conclusion that GlcA seems to be used more broadly in MOs, at least in Artiodactyla and Perissodactyla. This provides a third modality for negative charge in MOs, next to sialic acids and sulfation. In cows, GlcA also seems to be post-biosynthetically sulfated (27), potentially implying that the human natural killer-1 (HNK-1, CD57) epitope, important in immune regulation (68), could be found in MOs. Combined with our macrophage immunomodulation results and the paucity of known pathogens that could be inhibited by GlcA, we speculate that physiological functions of GlcA-containing MOs might relate to immune modulation, an important area for MOs (6), such as facilitating the establishment of the infant microbiome. However, we must caution that the setup presented here constitutes a cross-species assay, as GlcA-containing glycans have not yet been detected in human milk.

While for human MOs specific functions have been extensively investigated (59, 69, 70), functional roles of MO motifs or structures in most animals still remain elusive. So far, MO functions seem to fall into the broad categories of microbiome development, nutrition, anti-pathogen, and immunomodulation (4, 8, 71, 72, 73). Future work could investigate potential anti-pathogen functionality of the described structures, in cases where species-specific pathogens are known. Computational methods, predicting binding to pathogen glycan-binding proteins (74), and screening methods, such as glycan arrays (75), could shed light on functions of motifs such as the herein-discovered MO LacdiNAc. Another possible avenue lies in their function as prebiotics, especially as beta-glucuronidases seem prevalent in at least human microbiota (76), for which a microbiome analysis of the respective species will be key.

Our work demonstrates that MOs, especially non-human MOs, are substantially more diverse than currently appreciated. In multiple species, and multiple oligosaccharides within those species, we show that the MO repertoire contains more monosaccharides (GlcA) and more motifs (*e.g.*, LacdiNAc) than canonically assumed. We anticipate that further exploration of MOs will uncover further biochemical diversity. Our curated and measured dataset represents a currently exhaustive collection of what is known about MO biodiversity, and we anticipate that it will be used to produce new insights into glycobiology, MO biosynthesis, and breast milk function.

## Data and Code Availability

All code used here is available in the Python package glycowork version 0.6. Data curated or generated here can be found in the supplementary tables as well as stored as internal datasets within glycowork. The glycomics MS raw files have been deposited in the GlycoPOST database under the ID GPST000317 (https://glycopost.glycosmos.org/preview/197848368063b54edf7c2f6; code:7373).

## Supplemental data

This article contains supplemental data.

## Conflict of interest

The authors declare that they have no known competing financial interests or personal relationships that could have appeared to influence the work reported in this paper.
