# Supplementary material for: Breast Milk Oligosaccharides Contain Immunomodulatory Glucuronic Acid and LacdiNAc
Source: Mol Cell Proteomics. 2023 Aug 18;22(9):100635. doi: 10.1016/j.mcpro.2023.100635 (PMC10509713; doi:10.1016/j.mcpro.2023.100635)
Supplement: Supplemental Tables S1 and S2 and Figures S1–S6 [file mmc4.pdf]

Breast Milk Oligosaccharides Contain Immunomodulatory Glucuronic Acid and LacdiNAc

Chunsheng Jin[1,§], Jon Lundstrøm[2,§], Emma Korhonen[2], Ana S. Luis[3], Daniel Bojar[2,*]

[1]Proteomics Core Facility at Sahlgrenska Academy, University of Gothenburg, Gothenburg, Sweden

[2]Department of Chemistry and Molecular Biology, University of Gothenburg, Gothenburg, Sweden. Wallenberg Centre for Molecular and Translational Medicine, University of Gothenburg, Gothenburg, Sweden.

[3]Department of Medical Biochemistry and Cell Biology, University of Gothenburg, Gothenburg, Sweden.

[§]These authors contributed equally to this work

[*]Lead contact: Daniel Bojar, daniel.bojar@gu.se

Supplementary Table Legends

Supplementary Table S1. Relative abundances of characterized milk oligosaccharides. For all analyzed samples (different species as well as fractions including neutral, acidic, lactose-depleted, etc.), we provide the identified glycan structures and their accompanying relative abundance in a given sample. Further, retention time, composition, its sequence in IUPAC-condensed, and reducing mass of each structure is provided.

Supplementary Table S2. Curated dataset of milk glycan – species associations from the academic literature. Each row contains a glycan sequence and the species (and higher-level taxonomic group) in which it was identified in its milk, accompanied by the corresponding reference.

Supplementary Dataset S1. MS/MS spectra of all described glycans. For all 172 described glycan structures, we show representative MS/MS spectra, with annotated peaks. We also describe the *m/z* and charge at which the structure was identified, the species in which it was identified, and whether it presented an entirely novel sequence. The structure itself is shown via the Symbol Nomenclature for Glycans (SNFG).

Supplementary Figures

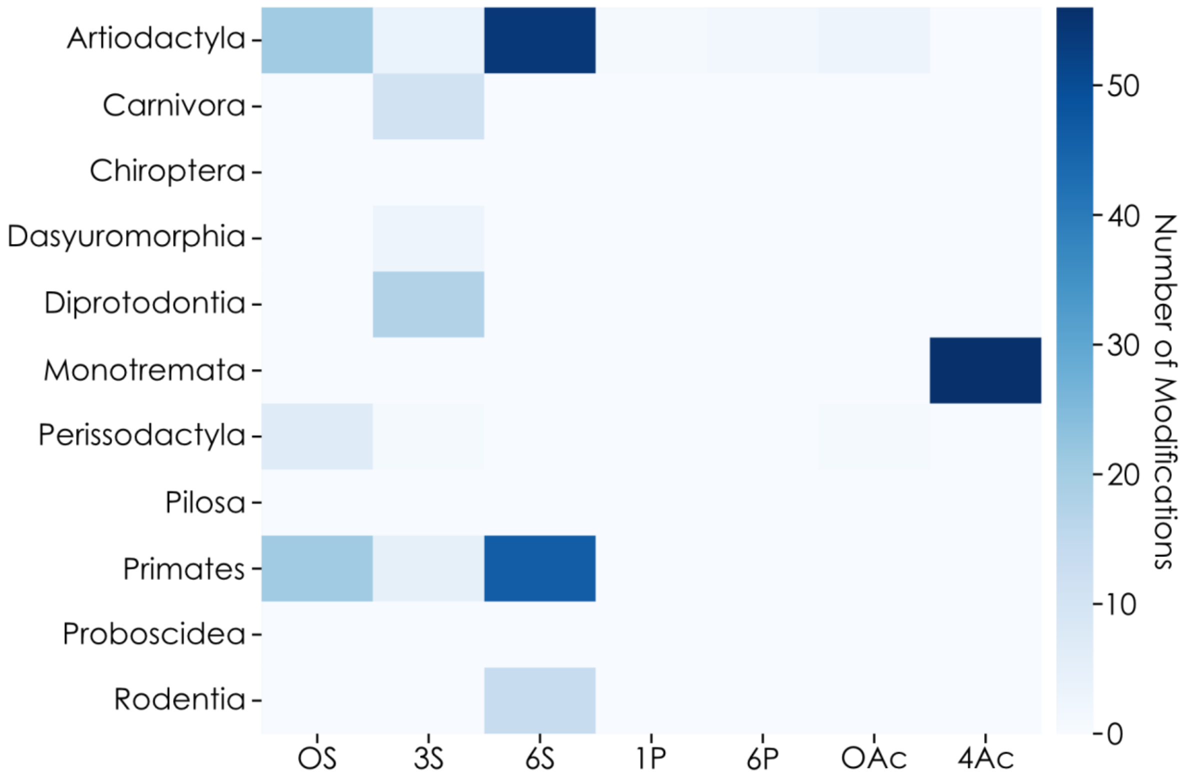

Supplementary Figure S1. Distribution of post-biosynthetic modifications across milk oligosaccharides. For all species with more than five milk oligosaccharides, we counted the occurrence of sulfation (OS, 3S, 6S), phosphorylation (1P, 6P), and acetylation (OAc, 4Ac) and summed them by taxonomic order, depicted as a heatmap.

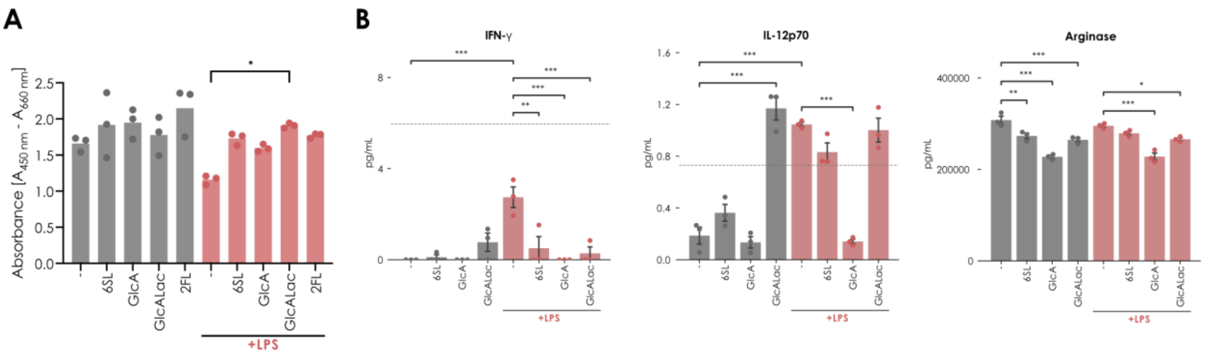

Supplementary Figure S2. Effect of glucuronylated milk glycans on viability and other cytokines. (A) XTT viability assay of THP-1 cells unstimulated (grey) or stimulated with LPS (red) in the absence or presence of various MO-derived glycan structures. **(B)** Measurement of cytokine concentration of IFN-γ, IL-12p70, and Arginase from the culture supernatant of THP-1 cells unstimulated (grey) or stimulated with LPS (red) in the absence or presence of various MO-derived glycan structures. The dashed line indicates the limit of detection as determined by the standard curve of each analyte. Significant differences were established via a one-way ANOVA with Tukey's multiple comparison test. ***, $p < 0.001$; **, $p < 0.01$; *, $p < 0.05$.

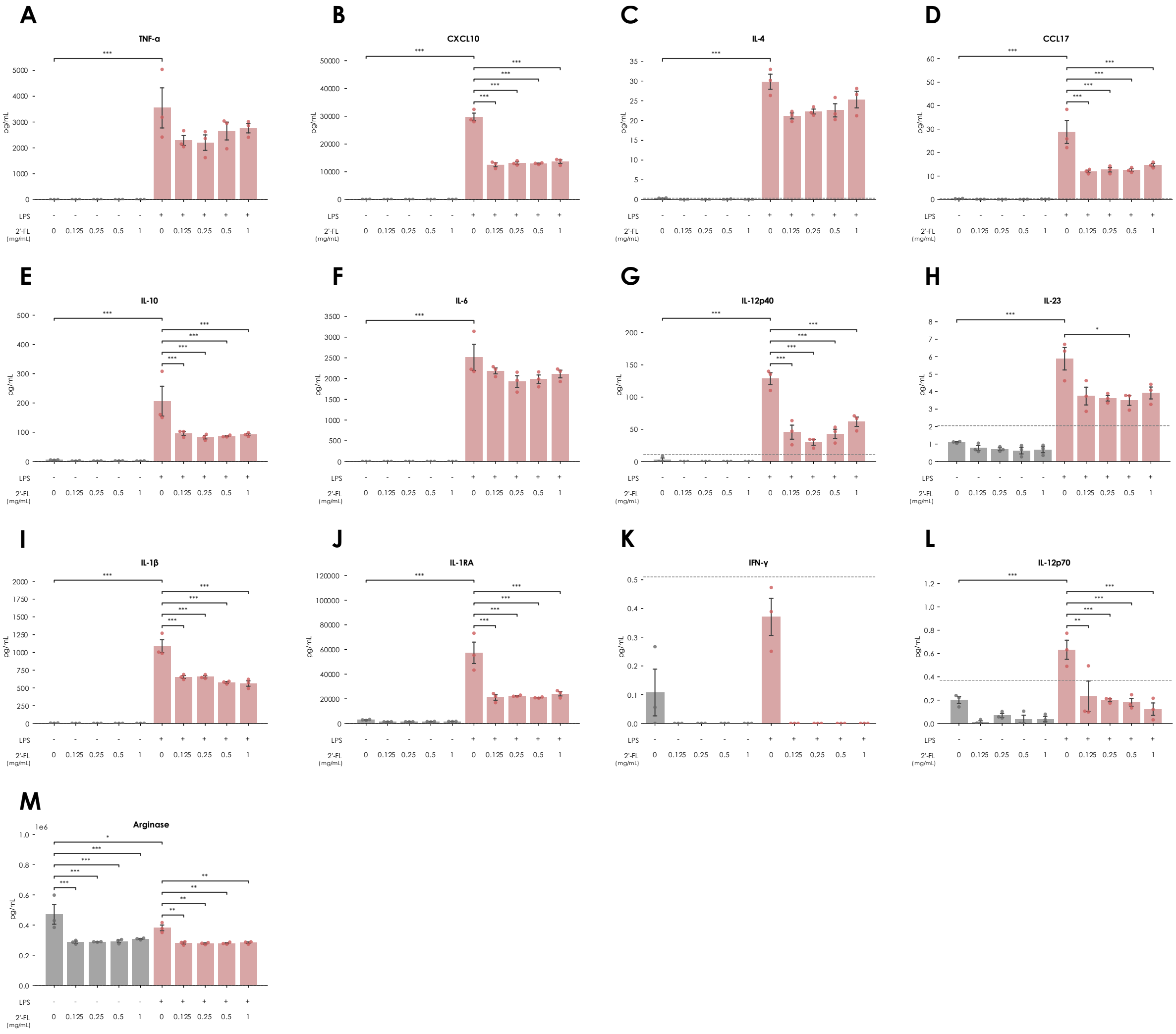

Supplementary Figure S3. Dose-dependent effect of 2'-FL on LPS-induced cytokine production. Quantification of cytokine concentrations of TNF-α (**A**), CXCL10 (**B**), IL-4 (**C**), CCL17 (**D**), IL-10 (**E**), IL-6 (**F**), IL-12p40 (**G**), IL-23 (**H**), IL-1β (**I**), IL-1RA (**J**), IFN-γ (**K**), IL-12p70 (**L**), and arginase (**M**) from the culture supernatant of THP-1 cells unstimulated (grey) or stimulated with LPS (red) in the absence or presence of varying concentrations of 2'-FL. The dashed line indicates the limit of detection as determined by the standard curve of each analyte. Significant differences were established via a one-way ANOVA with Tukey's multiple comparison test. ***, $p < 0.001$; **, $p < 0.01$; *, $p < 0.05$.

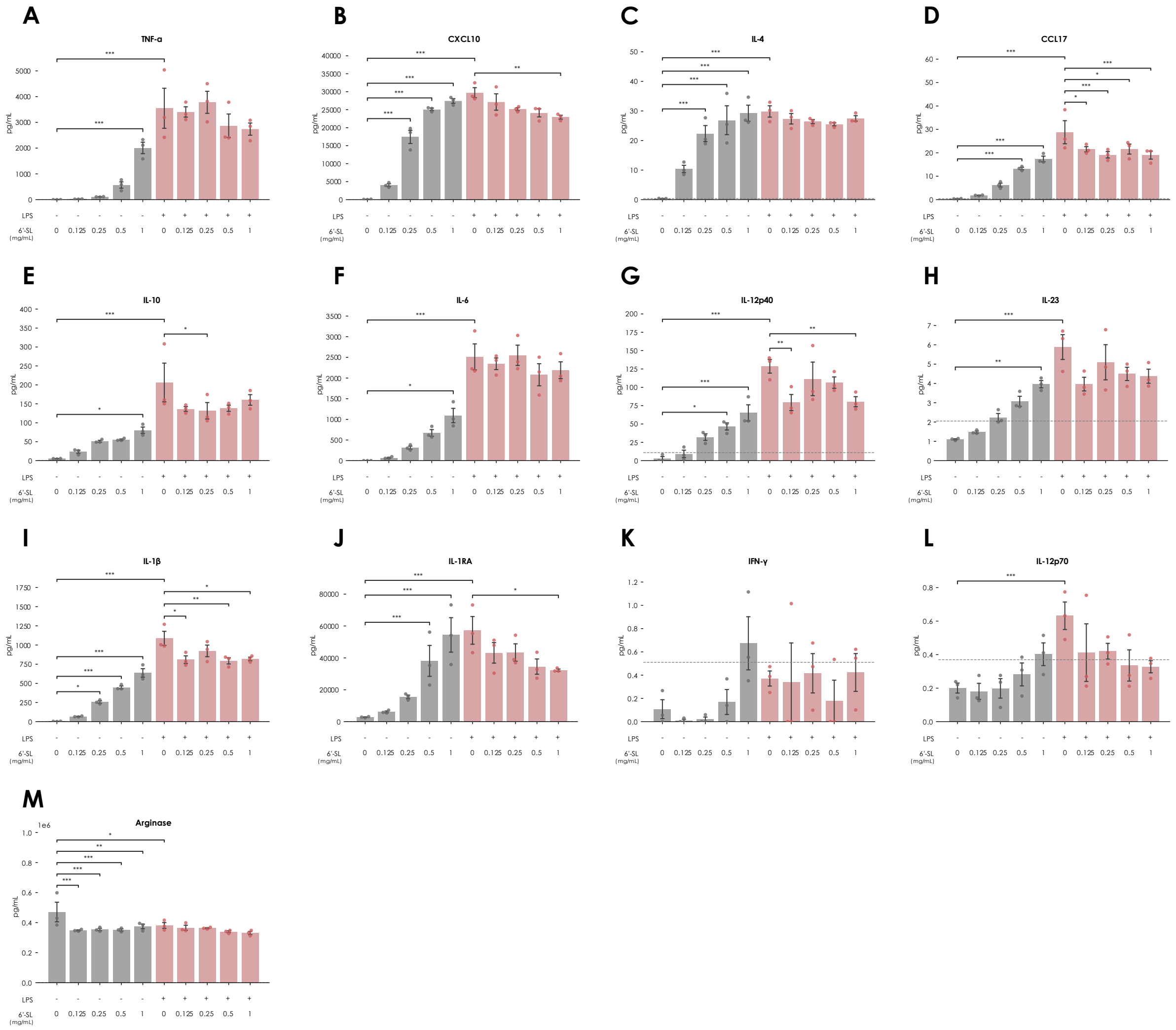

Supplementary Figure S4. Dose-dependent effect of 6'-SL on LPS-induced cytokine production. Quantification of cytokine concentrations of TNF-α (**A**), CXCL10 (**B**), IL-4 (**C**), CCL17 (**D**), IL-10 (**E**), IL-6 (**F**), IL-12p40 (**G**), IL-23 (**H**), IL-1β (**I**), IL-1RA (**J**), IFN-γ (**K**), IL-12p70 (**L**), and arginase (**M**) from the culture supernatant of THP-1 cells unstimulated (grey) or stimulated with LPS (red) in the absence or presence of varying concentrations of 6'-SL. The dashed line indicates the limit of detection as determined by the standard curve of each analyte. Significant differences were established via a one-way ANOVA with Tukey's multiple comparison test. ***, $p < 0.001$; **, $p < 0.01$; *, $p < 0.05$.

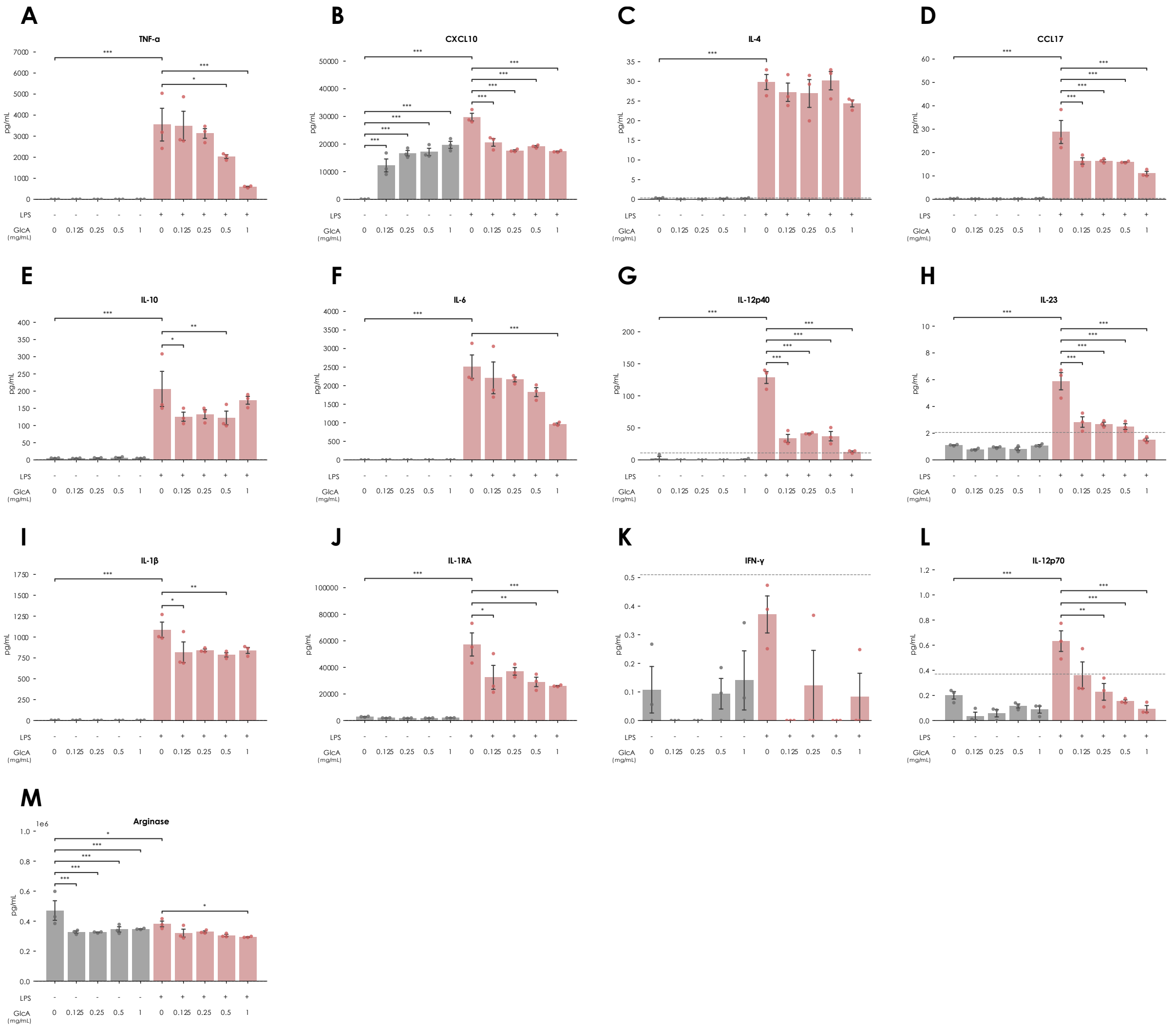

Supplementary Figure S5. Dose-dependent effect of GlcA on LPS-induced cytokine production. Quantification of cytokine concentrations of TNF-α (**A**), CXCL10 (**B**), IL-4 (**C**), CCL17 (**D**), IL-10 (**E**), IL-6 (**F**), IL-12p40 (**G**), IL-23 (**H**), IL-1β (**I**), IL-1RA (**J**), IFN-γ (**K**), IL-12p70 (**L**), and arginase (**M**) from the culture supernatant of THP-1 cells unstimulated (grey) or stimulated with LPS (red) in the absence or presence of varying concentrations of GlcA. The dashed line indicates the limit of detection as determined by the standard curve of each analyte. Significant differences were established via a one-way ANOVA with Tukey's multiple comparison test. ***, $p < 0.001$; **, $p < 0.01$; *, $p < 0.05$.

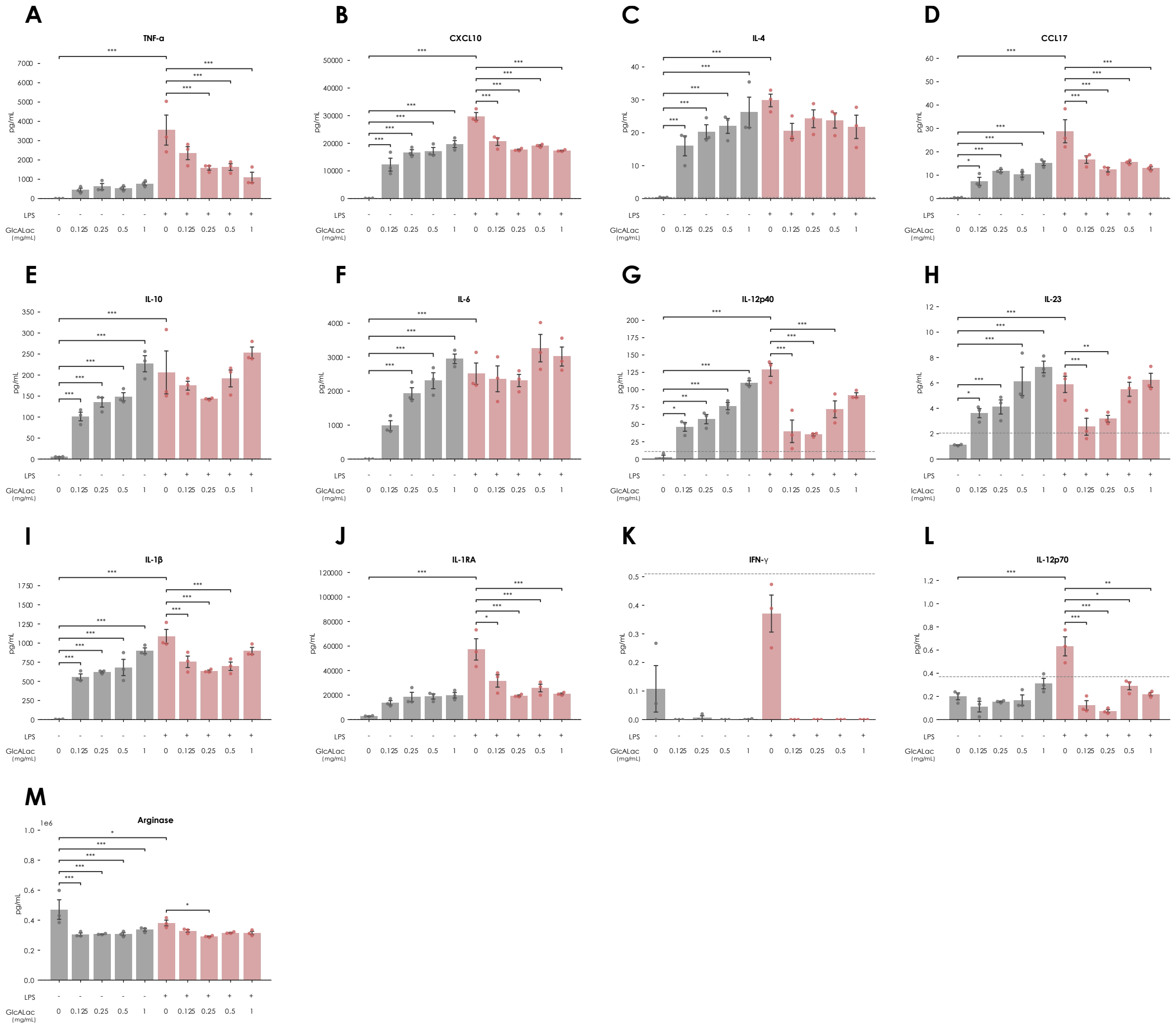

Supplementary Figure S6. Dose-dependent effect of GlcALac on LPS-induced cytokine production. Quantification of cytokine concentrations of TNF-α (**A**), CXCL10 (**B**), IL-4 (**C**), CCL17 (**D**), IL-10 (**E**), IL-6 (**F**), IL-12p40 (**G**), IL-23 (**H**), IL-1β (**I**), IL-1RA (**J**), IFN-γ (**K**), IL-12p70 (**L**), and arginase (**M**) from the culture supernatant of THP-1 cells unstimulated (grey) or stimulated with LPS (red) in the absence or presence of varying concentrations of GlcALac. The dashed line indicates the limit of detection as determined by the standard curve of each analyte. Significant differences were established via a one-way ANOVA with Tukey's multiple comparison test. ***, $p < 0.001$; **, $p < 0.01$; *, $p < 0.05$.
